# Parametric Finite Element Modeling of Cold Roll-Beating Forming of Explosively Bonded Fe/Al Layered Composites Incorporating an Equivalent Bonding Layer

**DOI:** 10.3390/ma19214745

**Published:** 2026-11-08

**Authors:** Limu Cui, Yan Li, Mingshun Yang, Qilong Yuan, Long Li

**Affiliations:** School of Mechanical Engineering, Xi’an University of Technology, Xi’an 710048, China; jyxy-ly@xaut.edu.cn (Y.L.); yangmingshun@xaut.edu.cn (M.Y.); yuanql@xaut.edu.cn (Q.Y.); lilong678@stu.xaut.edu.cn (L.L.)

**Keywords:** cold roll-beating forming, Fe/Al layered composites, explosive welding, equivalent bonding layer, finite element modeling, interfacial characterization

## Abstract

A parametric finite element (FE) framework incorporating a finite-thickness equivalent bonding layer is developed for cold roll-beating of explosively bonded Fe/Al layered composites. The equivalent layer thickness is identified from interfacial characterization and the through-thickness microhardness gradient, while its quasi-static and strain-rate-dependent response is determined from nanoindentation, compression, split Hopkinson pressure bar tests, and inverse FE identification. The resulting properties are assigned to a three-layer FE model comprising the steel clad, equivalent bonding layer, and aluminum substrate. Experimental validation gives mean absolute relative errors of 8.35% and 6.20% for forming-force prediction in the spindle-speed and feed-speed series, respectively, and a mean profile normalized root-mean-square error (NRMSE) of 3.27% across six validation conditions. These errors are consistently lower than those of the simplified bilayer model. With increasing spindle speed, the peak forming force first increases slightly and then decreases, while the tooth root deepens and material pile-up increases. Increasing feed speed from 100 to 225 mm/min raises the predicted peak forming force from 13.62 to 14.98 kN but produces a shallower profile. These different trends indicate that peak force reflects local deformation resistance, whereas the final profile is governed mainly by plastic deformation accumulated over successive engagements.

## 1. Introduction

Lightweight design has become an important strategy for improving the performance and energy efficiency of transmission systems. Reducing the mass of moving components can decrease inertial loads and energy consumption, while critical transmission components such as gears must still satisfy stringent requirements for load-carrying capacity, structural stiffness, and dimensional accuracy [1,2,3]. Lightweighting is commonly achieved through structural optimization, material substitution, or multi-material design. Although multi-material components can exploit the complementary properties of different constituents, conventional joining methods such as bolting, riveting, and welding may introduce discrete joints and local discontinuities in structure or properties, while also imposing requirements on joint reliability and assembly accuracy [4,5,6,7,8]. Dissimilar layered metallic composites provide an alternative approach by integrating different constituent materials into a continuous load-bearing structure through interfacial bonding, thereby combining complementary material properties while avoiding some of the structural and assembly constraints associated with discrete joints [9,10,11,12]. In an explosively bonded Fe/Al layered composite, for example, the steel clad provides high surface load-carrying capacity, whereas the low-density aluminum substrate contributes to overall weight reduction [13]. However, the pronounced mismatch in mechanical properties between the constituent layers can lead to substantial through-thickness deformation incompatibility during the forming of complex profiles [14]. Cold roll-beating is an incremental forming process in which periodic local contact with the roller produces successive small increments of plastic deformation that accumulate to form the target profile, making it attractive for the near-net-shape forming of complex-profile components [15]. During cold roll-beating of heterogeneous layered composites, the mechanical mismatch among the steel clad, aluminum substrate, and bonding region gives rise to distinct deformation responses under repeated local loading, resulting in more complex interlayer load transfer, plastic flow, and overall forming behavior than in homogeneous materials.

Explosive welding produces a characteristic wavy Fe/Al interface through high-velocity collision and severe local plastic deformation, often accompanied by elemental mixing and localized melting and resolidification [16,17,18]. The resulting bonding region therefore contains microstructurally distinct features rather than a single geometric discontinuity. Previous observations have also shown that microstructural gradients and spatial variations in mechanical properties extend over a finite distance across explosively bonded interfaces [19,20,21]. During cold roll-beating, this region lies directly in the load-transfer path between the steel clad and aluminum substrate, so its response can influence the local stress–strain state, plastic flow, and profile development. A component-scale forming model should therefore retain both the finite spatial extent of the bonding region and a mechanical response distinct from those of the adjoining materials.

Two commonly used modeling strategies capture different aspects of an explosively bonded interface. A zero-thickness formulation is computationally efficient and can describe interfacial bonding, separation, and damage when an appropriate interface law is available [22,23,24]. However, because the interface has no volume, deformation within a finite bonding region and its through-thickness variation cannot be represented directly. Explicitly resolving the wavy interface and associated microstructural features avoids this geometric simplification, but requires much finer geometric discretization and material partitioning [25]. The resulting computational cost becomes substantial for cold roll-beating simulations involving repeated contact and multiple process conditions. In the present work, the bonding region is therefore homogenized as a finite-thickness equivalent layer. This representation retains its volumetric contribution at the component scale without resolving individual interfacial features and allows experimentally characterized interface-scale information to be introduced into the forming model [26,27]. The remaining requirement is to determine the equivalent-layer thickness and constitutive response experimentally and assign them consistently in the FE model [27,28,29].

Previous studies of cold roll-beating have focused predominantly on homogeneous metallic materials, addressing process kinematics, periodic contact and local loading, forming forces, material flow, and the effects of process parameters on forming behavior [15,30,31,32,33]. Related investigations of gear and spline rolling have further highlighted the influence of material properties and plastic flow on tooth-profile accuracy [34]. By contrast, studies on the forming of dissimilar-material layered systems have mainly considered constituent-property mismatch, interlayer constraint, interface damage, and deformation compatibility [14,35,36,37]. In numerical simulations, interlayer bonding is commonly represented by ideal interfaces [22], cohesive-zone formulations [38], or explicit intermediate layers [28]. Zero-thickness formulations are well suited to describing interfacial bonding, separation, and damage when an appropriate interface law is available [22,39], whereas finite-thickness intermediate layers can additionally represent volumetric deformation and an effective material response distinct from those of the adjoining constituents [27,28,29]. Cold roll-beating, however, is characterized by repeated local dynamic loading, strain-rate-dependent material response, and the progressive accumulation of small plastic deformation increments [30,40,41], producing load-transfer and material-flow characteristics that differ from those in conventional bulk forming. Existing studies have separately addressed the experimental characterization of explosively bonded interfaces [16,17,19,20] and finite-thickness interphase or transition-layer modeling [27,28,29].

However, the quantitative linkage between experimentally identified bonding-region characteristics and their finite-thickness equivalent representation in component-scale forming simulations remains limited. Moreover, the influence of such a representation on predicted forming forces, final profiles, and process-parameter trends has received limited systematic assessment.

In this study, an experimentally informed parametric finite element framework is developed for cold roll-beating of explosively bonded Fe/Al layered composites. The principal methodological contribution lies in linking the experimentally characterized mechanical heterogeneity of the bonding region to its finite-thickness equivalent representation in a component-scale forming model. Specifically, microstructural and elemental characterization is performed to establish the heterogeneous nature of the bonding region, while its mechanically affected extent is determined from the through-thickness microhardness gradient. Nanoindentation, quasi-static compression, and split Hopkinson pressure bar (SHPB) tests, together with inverse FE identification, are employed to determine the effective elastoplastic and strain-rate-dependent constitutive response of the equivalent bonding layer. The resulting three-layer representation incorporates the volumetric contribution of the bonding region under the assumption of perfect interlayer bonding. This experimentally informed material representation is further integrated into an automated parametric FE framework that links roller geometry, process kinematics, layered workpiece geometry, material properties, and analysis settings. The predictive capability of the proposed approach is evaluated against experimental stable-stage forming forces and final tooth profiles and compared with that of a simplified bilayer model. The validated model is subsequently used to investigate the effects of spindle speed and feed speed, as well as the associated layerwise stress, plastic strain, and displacement responses under repeated local loading.

## 2. Parametric Modeling Strategy for Cold Roll-Beating

### 2.1. Forming Principle and Kinematic Description

**Figure 1:**
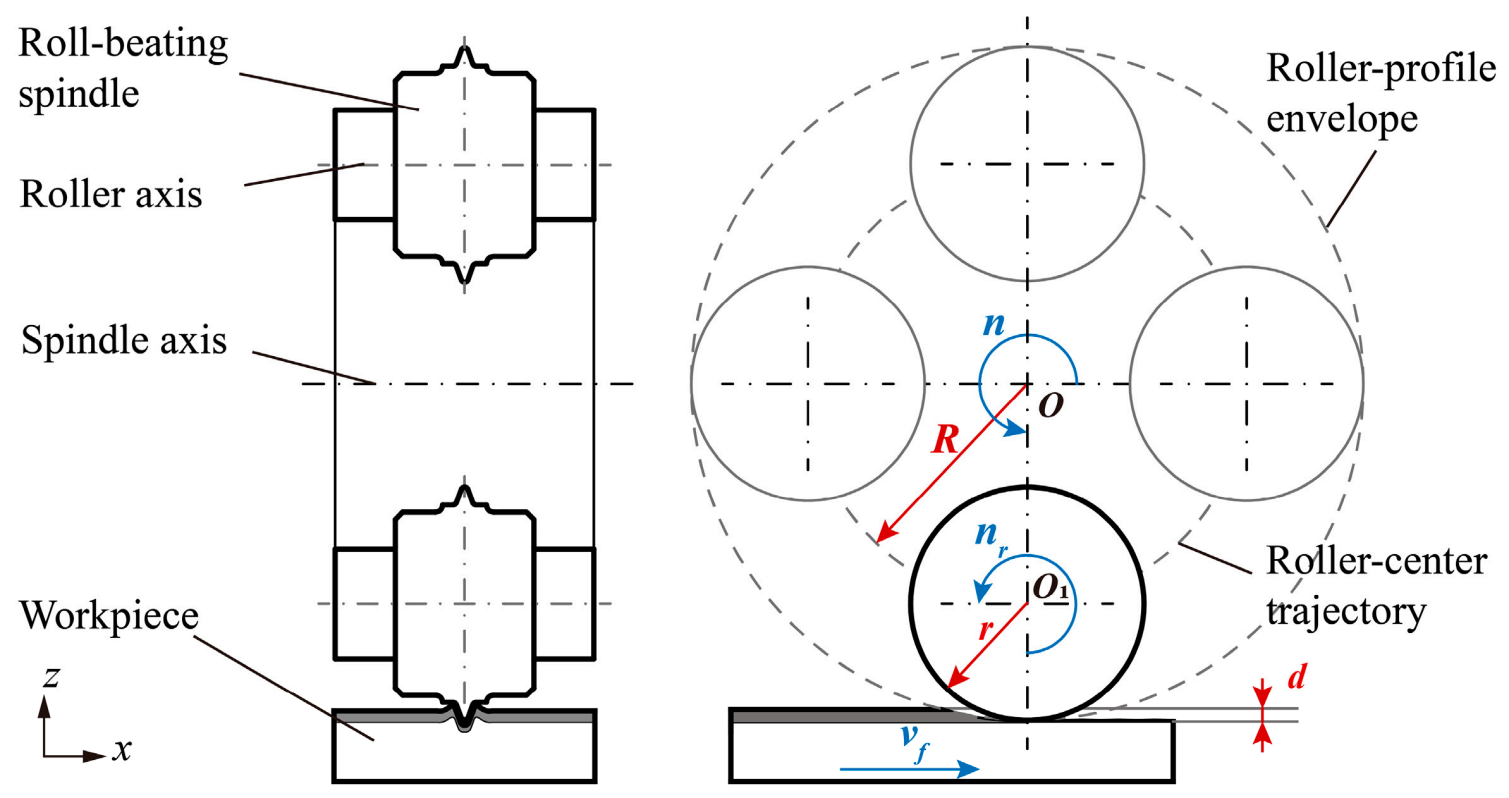
Kinematic relationships and control parameters for cold roll-beating of explosively bonded layered composites.

Figure 1 shows the kinematic configuration and principal control parameters of cold roll-beating. The roll-beating roller revolves about the spindle center *O* along a circular path of radius R as the spindle rotates at speed n. Its outer profile radius is r, and rotation about the roller axis O1 remains free, allowing passive self-rotation to develop through frictional contact with the workpiece. The workpiece simultaneously advances at a constant feed speed *v_f_*. The nominal roll-beating depth d is defined by the relative position of the spindle center with respect to the initial workpiece surface. Consequently, the combined orbital motion of the roller and translational motion of the workpiece produce successive local engagements along the feed direction, through which plastic deformation accumulates progressively [32].

**Figure 2:**
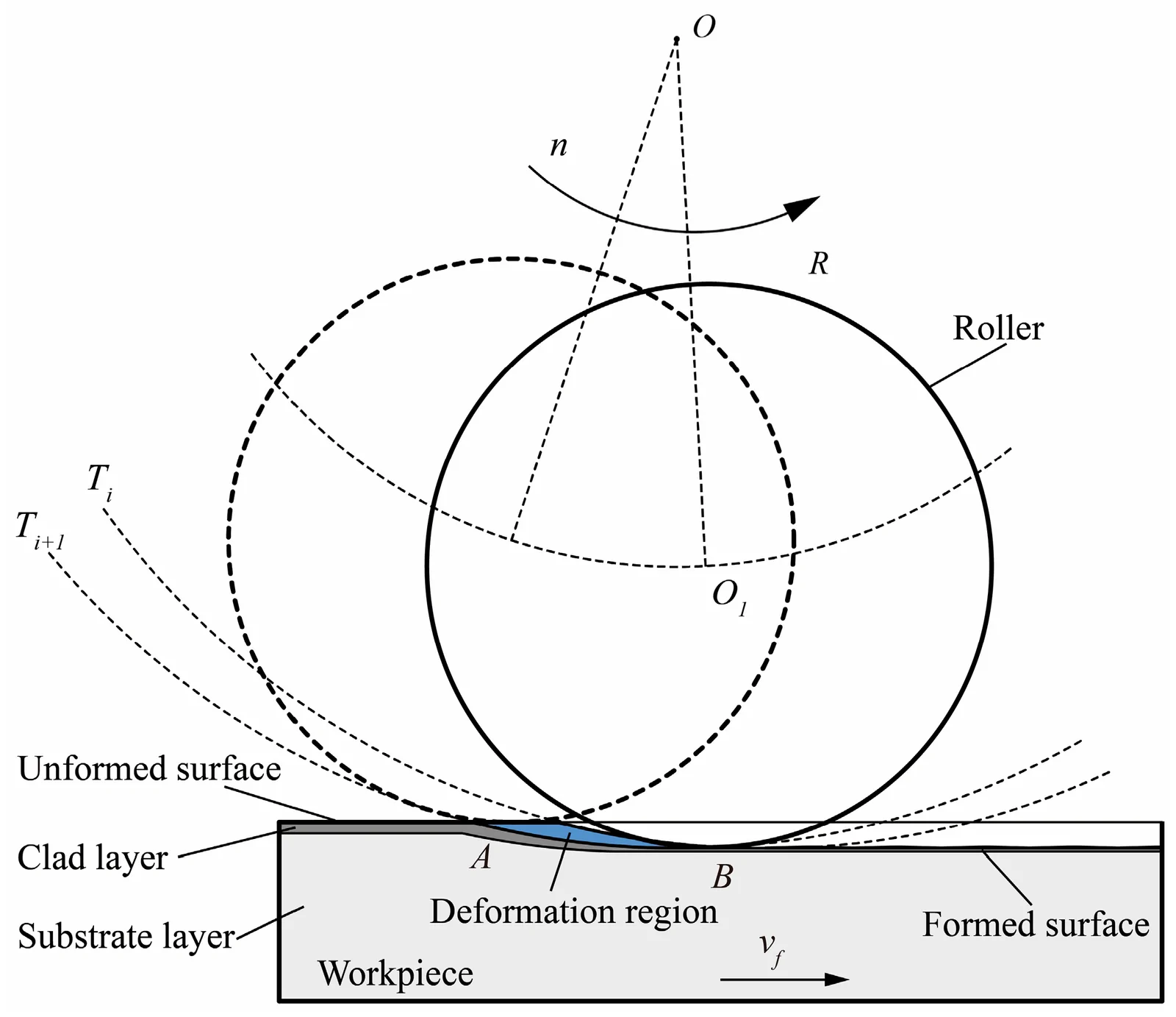
Schematic of local deformation during successive roll-beating engagements.

Figure 2 illustrates the deformation sequence during two successive roll-beating engagements. *T_i_* denotes the trajectory of the completed *i*th engagement, and *T_i_*_+1_ corresponds to the subsequent engagement. Along *T_i_*_+1_, contact begins at point *A*, after which continued spindle rotation drives the roller into the steel clad. Plastic deformation and material redistribution then develop within the evolving contact region as the roller advances along its trajectory. Contact ends at point *B*, completing the (*i* + 1)th engagement and leaving a locally deformed surface that is subsequently modified by the following engagements.

In the Fe/Al layered composite, the contact load is transferred from the steel clad through the bonding region to the aluminum substrate. The mechanical mismatch among the three regions affects the through-thickness deformation and the compatibility of the bonded layers. As the workpiece feeds forward, deformation produced by successive engagements overlaps along the feed direction, and the accumulated local deformation gradually forms the final profile.

### 2.2. Parametric Model Construction

The kinematic relationships defined in Section 2.1 are incorporated into a parametric FE model in which the tool configuration, roller profile, process conditions, and layered workpiece geometry are specified independently. Each input parameter is linked to the corresponding geometric or analysis definition, so that changes in the prescribed values are transferred directly to the generated FE model.

Four groups of inputs are used in the model: tool configuration, roller profile, process conditions, and layered workpiece geometry. The tool configuration is specified by the roller orbital radius *R* and its installation position relative to the spindle center, which determine the prescribed roller trajectory. The working profile of the roller is controlled by *r*, *m*, the filet radii *r*_1_–*r*_4_, and the remaining tooth-profile dimensions. The process inputs are the spindle speed *n*, feed speed *v_f_*, and nominal roll-beating depth *d*. The layered workpiece is defined by length *L*, width *B*, and total thickness *H*, together with the thicknesses of the steel clad *h_c_*, equivalent bonding layer *h_b_*, and aluminum substrate *h_s_*, where *H* = *h_c_* + *h_b_* + *h_s_*.

**Figure 3:**
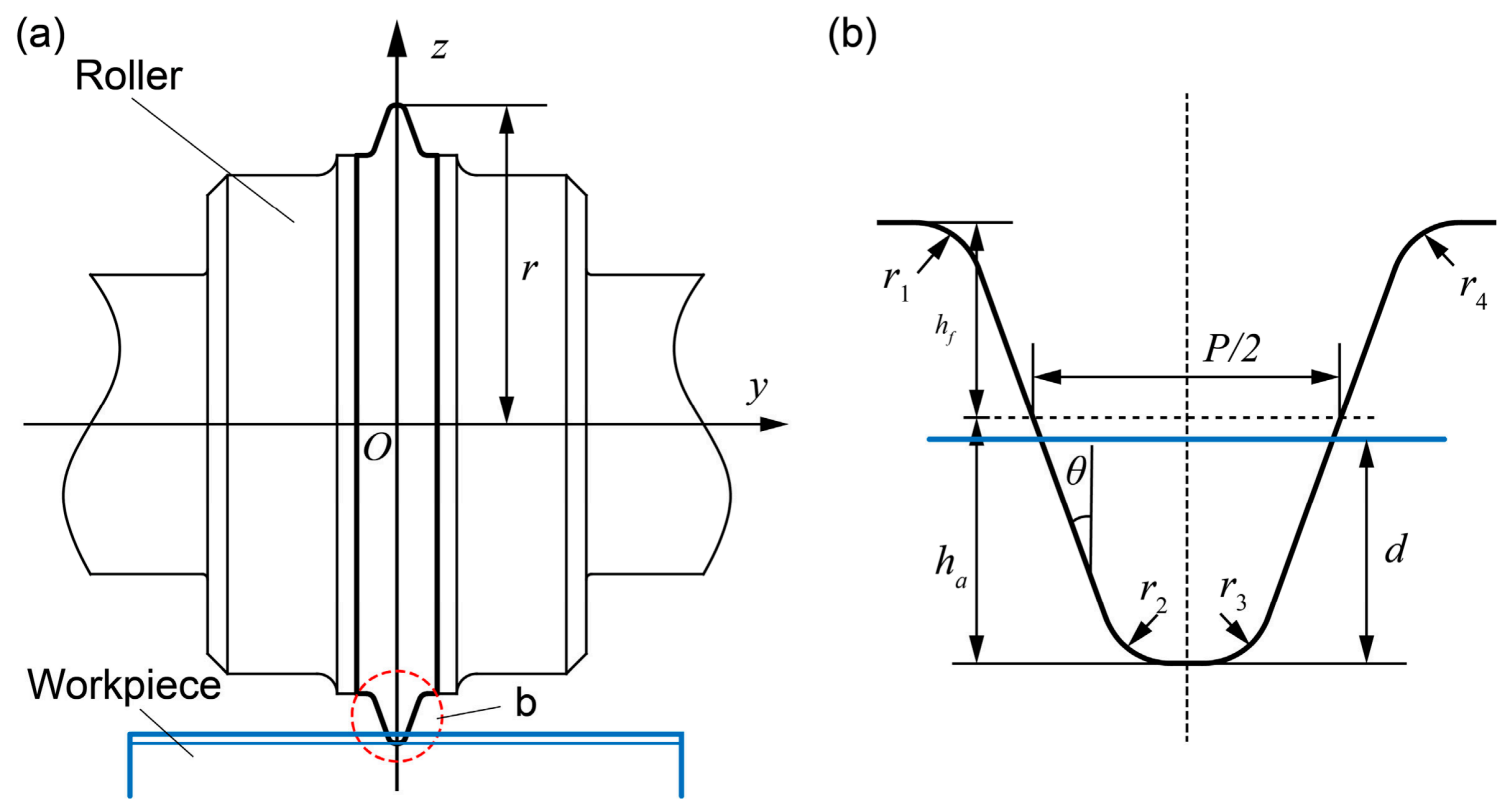
Schematic of the roller configuration and tooth-profile geometry: (**a**) roller–workpiece configuration; (**b**) roller tooth-profile parameters.

The roll-beating roller is made of 20CrMnTi steel, and its working profile is constructed as the geometric complement of the target tooth space, so that its protruding regions define the material displacement required to generate the prescribed tooth profile. The principal geometric dimensions of the roller profile are determined from the module, addendum coefficient, clearance coefficient, and filet radii of the target geometry, as illustrated in Figure 3. The corresponding geometric relationships are defined by *h_a_* = (c* + h_a_*)*m*, *h_f_* = h_a_* *m*, and *p* = *πm*, while the filet radii satisfy *r*_1_ = *r*_2_ = *r*_3_ = *r*_4_. These explicit relationships allow the roller profile to be generated automatically from the prescribed geometric inputs.

**Figure 4:**
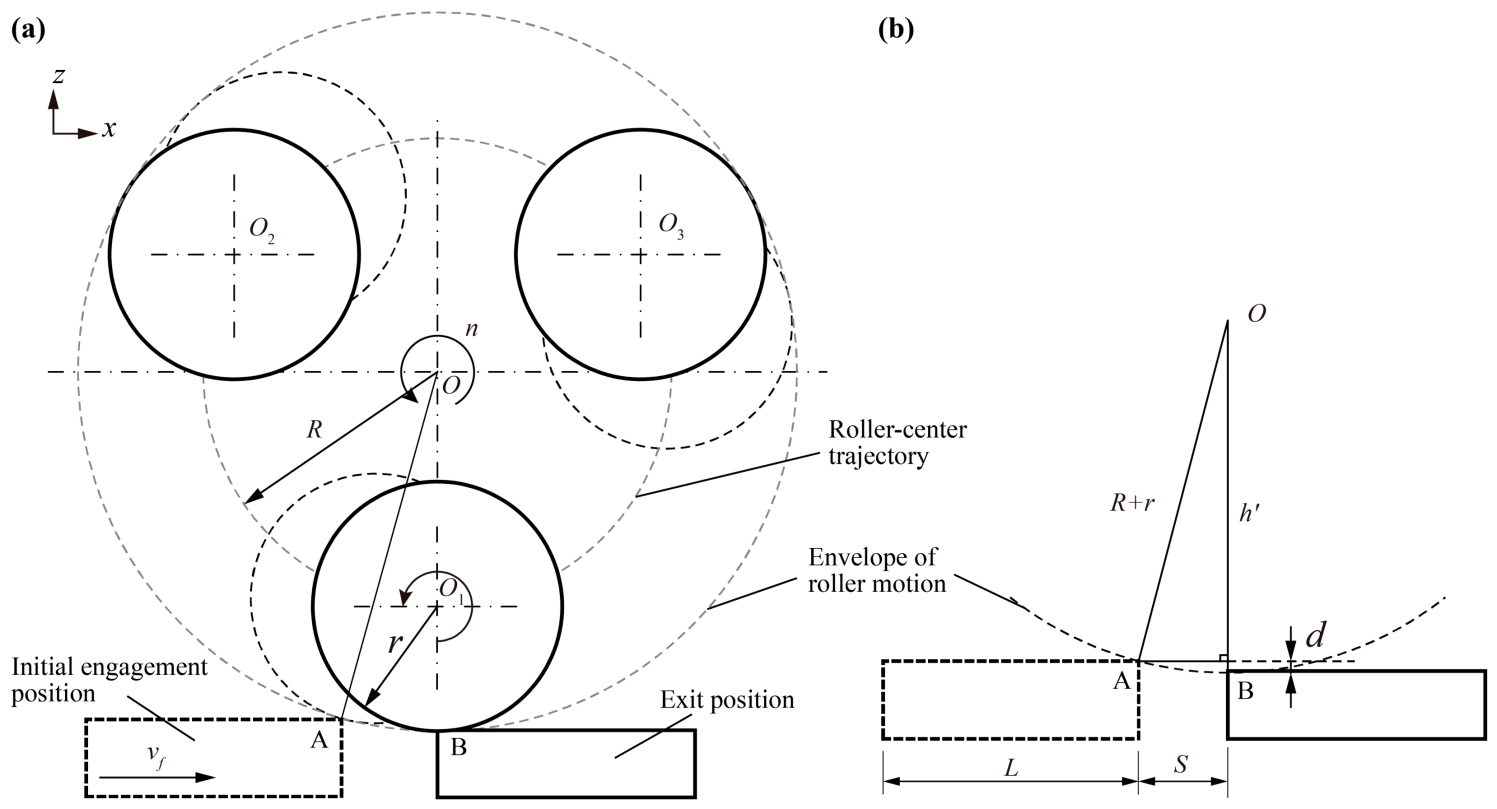
Geometric relationships used to determine the analysis-step duration in cold roll-beating: (**a**) roller–workpiece positions at initial engagement and final disengagement; (**b**) geometric definition of the horizontal distance *S*.

In addition to geometric parameterization, the duration of the explicit analysis is determined automatically from the prescribed roller–workpiece motion. As shown in Figure 4, the roller center revolves periodically about the spindle center, while the workpiece advances at a constant feed speed *v_f_*. The envelope radius of the roller motion is *R* + *r*. Let *h’* denote the distance from the spindle center to the initial workpiece surface. Its relationship with the nominal roll-beating depth d is given by

*h*^′^ = *R* + *r* − *d* (1)

The horizontal distance *S* between the intersection of the roller-motion envelope with the initial workpiece surface and the projection of the spindle center onto that surface is expressed as

(2) $$S=\sqrt{{\left(R+r\right)}^{2}-h^{\prime 2}}$$

The initial engagement and final disengagement positions are denoted by position A and position B, respectively. Initial engagement occurs when the leading edge of the workpiece intersects the roller-motion envelope at position A, whereas final disengagement occurs when the trailing edge reaches the lowest point of the envelope at position B. The corresponding horizontal displacement of the workpiece is *L* + *S*, giving the total analysis-step duration as

(3) $$t=\frac{L+S}{v_{f}}$$

where *t* denotes the total duration of the analysis step.

The resulting step duration covers the roller motion from initial engagement to final disengagement and therefore includes the stable forming stage between them. Because *t* is evaluated directly from the prescribed geometry and feed speed, the analysis duration is updated automatically whenever the corresponding model inputs are changed. The same parameter links are used during subsequent layer partitioning, meshing, contact definition, and boundary-condition assignment.

### 2.3. Refined Layered Modeling of the Explosively Bonded Fe/Al Layered Composite Based on Interfacial Characterization

**Figure 5:**
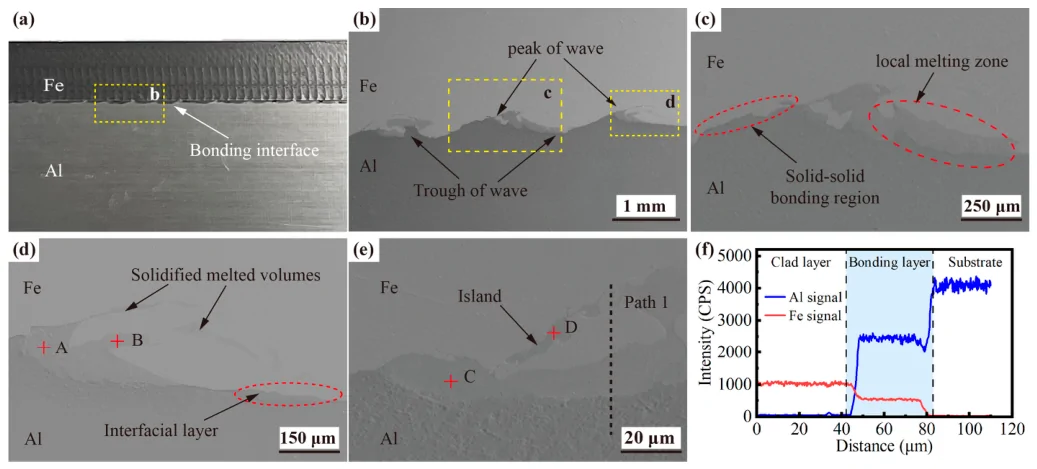
Microstructural and compositional characterization of the explosively bonded Fe/Al interface: (**a**) macroscopic cross-section of the steel–aluminum composite; (**b**) wavy Fe/Al interface; (**c**) locally melted and solid–solid bonded regions; (**d**) resolidified melt regions and interfacial layer; (**e**) island-like morphology and energy-dispersive X-ray spectroscopy (EDS) analysis locations; and (**f**) EDS line-scan profiles along Path 1 in (**e**).

To characterize the layered architecture and bonding-region features of the explosively bonded Fe/Al laminate, the interfacial microstructure and elemental distribution were examined using a scanning electron microscope (SEM; MERLIN Compact, Carl Zeiss Microscopy GmbH, Jena, Germany), as shown in Figure 5. The macroscopic cross-section in Figure 5a reveals a continuous bonding region between the steel clad and aluminum substrate. Further SEM observations in Figure 5b–e show a pronounced wavy interface with several characteristic local features, including wave crests and troughs, locally melted regions, solid–solid bonded regions, resolidified melt regions, and island-like morphologies. Together, these features show that the Fe/Al bonding region produced by explosive welding is structurally heterogeneous, reflecting severe local deformation as well as localized melting and resolidification [16,17,18]. The observed variations in interfacial morphology highlight the limitations of characterizing the three-dimensional interface from two-dimensional cross-sectional observations alone.

To examine the local elemental distribution across the interface, energy-dispersive X-ray spectroscopy (EDS) point analyses were conducted at representative locations A–D in Figure 5d,e, together with an EDS line scan along Path 1 in Figure 5e. As shown in Figure 5f, the Fe signal progressively decreases while the Al signal increases along the interface-normal direction from the steel clad toward the aluminum substrate, indicating a compositionally graded region in which Fe and Al signals coexist. The EDS point-analysis results summarized in Table 1 further show substantial concentrations of both Fe and Al at locations A–D, with Fe contents ranging from 34.79 to 50.21 at.% and Al contents from 46.93 to 63.47 at.%. Combined with the microstructural observations, these results reveal pronounced local compositional heterogeneity within the resolidified regions, interfacial layer, and island-like structures, which may result from local material mixing, elemental transport, and interfacial reactions during explosive welding [17,19].

On this basis, the bonding region is represented in the component-scale FE model as a finite-thickness equivalent bonding layer rather than a zero-thickness interface. Together with the steel clad and aluminum substrate, this treatment yields the three-layer representation used in the subsequent simulations. The equivalent layer thickness and constitutive response are determined from the experimental characterization described below.

**Figure 6:**
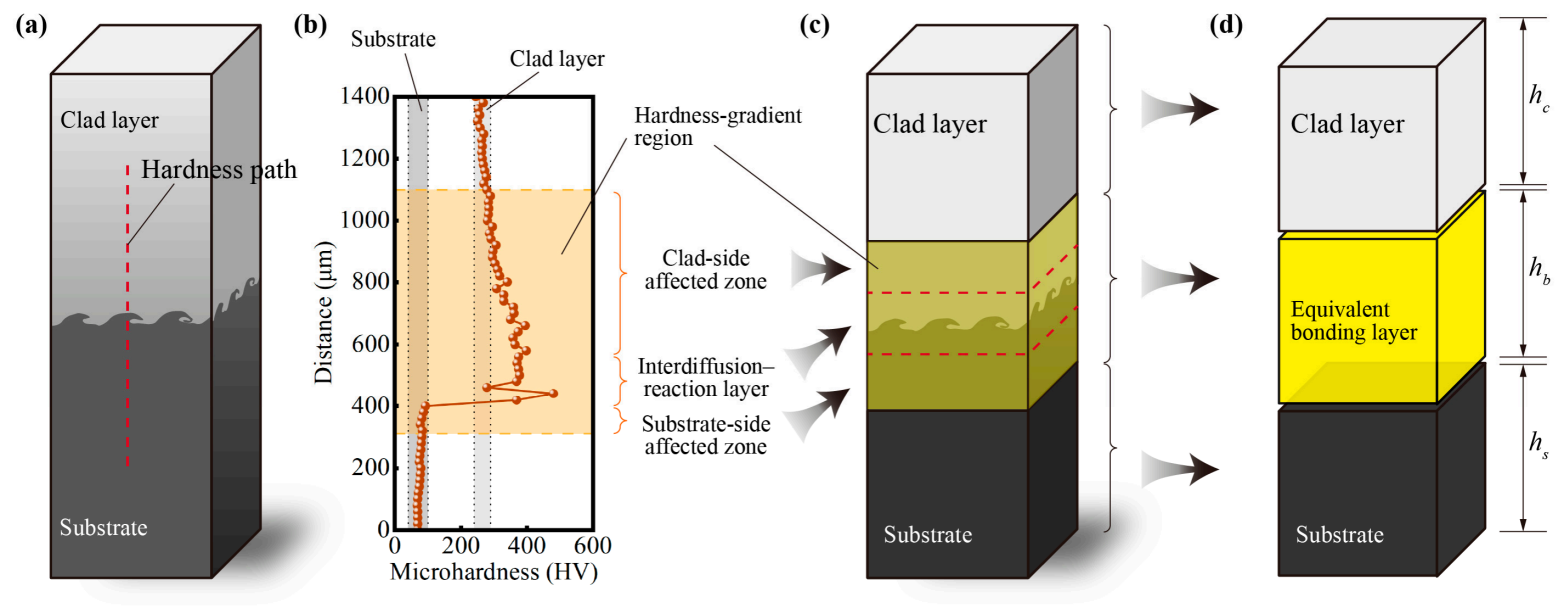
Definition of the equivalent bonding layer and refined layered FE representation of the explosively bonded Fe/Al composite based on the microhardness gradient. (**a**) Initial layered structure and hardness measurement path; (**b**) through-thickness microhardness profile used to identify the hardness-gradient region; (**c**) physical interpretation of the bonding region in terms of the clad-side deformation-affected zone, interdiffusion–reaction region, and substrate-side deformation-affected zone; and (**d**) idealized three-layer FE model consisting of the steel clad, equivalent bonding layer, and aluminum substrate, with *h_c_*, *h_b_*, and *h_s_* denoting their respective thicknesses.

The geometric extent of the equivalent bonding layer was identified from the microhardness distribution measured normal to the interface using a microhardness tester (HM-210C, Mitutoyo Corporation, Kawasaki, Japan), as illustrated in Figure 6. Figure 6a shows the initial layered configuration and the microhardness measurement path, while Figure 6b presents the corresponding through-thickness hardness profile. The hardness near the interface deviates markedly from the stable values measured in the adjoining bulk regions and shows a clear spatial variation, indicating that welding-induced plastic deformation, microstructural evolution, and compositional gradients collectively modify the local mechanical response. Similar variations in local mechanical properties near explosively welded Al/steel interfaces have been reported in previous studies [21]. Notably, the measured hardness variation extends into the adjoining constituent materials, indicating that the local mechanical response differs from that of the corresponding bulk material even within the same constituent. Consequently, explicitly representing the wavy interface between two homogeneous parent materials would not, by itself, account for the welding-induced spatial variation in mechanical properties.

The microhardness values measured in the unaffected steel clad and aluminum substrate were first statistically analyzed to establish their respective baseline hardness levels and characteristic fluctuations. The corresponding hardness values were 206.64 ± 15.53 HV and 56.07 ± 4.37 HV (mean ± standard deviation), respectively. To account for the inherent hardness scatter of the parent materials, the upper reference limits were defined as the mean plus one standard deviation, giving 222.17 HV for the steel clad and 60.44 HV for the aluminum substrate. These material-specific thresholds were adopted to identify regions of elevated hardness near the bonding interface.

Considering the spatial heterogeneity of the bonding region, microhardness distributions measured along multiple paths were evaluated in a common spatial coordinate system. The outermost boundaries of the regions exceeding the corresponding reference limits were used to define the spatial envelope of the mechanically affected region. Based on the identified spatial envelope, the equivalent bonding layer thickness *h_b_* was determined to be approximately 0.8 mm. This value represents the extent of the mechanically affected region identified from the hardness gradient, rather than the physical thickness of the metallurgical reaction layer.

Figure 6c further resolves the actual bonding region into a clad-side deformation-affected zone, a central interdiffusion–reaction region, and a substrate-side deformation-affected zone. The two outer zones are primarily associated with severe local plastic deformation and the accompanying microstructural evolution induced by explosive welding, whereas the central region is mainly characterized by compositional gradients, local material mixing, and possible interfacial reactions. This subdivision highlights the spatial heterogeneity of the bonding region before it is homogenized into the equivalent bonding layer used in the component-scale FE model.

A similar finite-thickness representation was adopted by Li et al. [28], who introduced an equivalent transition layer with independently estimated effective properties into a laser-bending FE model of a stainless steel/carbon steel laminated plate. In the present study, the equivalent representation is used to describe the effective elastoplastic and strain-rate-dependent response of the Fe/Al bonding region. This treatment does not imply that the actual bonding region is mechanically uniform; rather, its unresolved local variations are represented by an effective constitutive response for macroscopic forming analysis.

Although these subregions describe the underlying microstructural and mechanical heterogeneity of the actual bonding region, they are homogenized at the component scale into a single equivalent bonding layer with effective material properties. As shown in Figure 6d, the steel clad, equivalent bonding layer, and aluminum substrate are assigned thicknesses *h_c_*, *h_b_*, and *h_s_*, respectively, thereby defining a three-layer geometric model that explicitly retains the finite thickness of the bonding region. The two boundaries adjoining the equivalent layer are effective material-partition boundaries, rather than direct geometric representations of the actual Fe/Al metallurgical interface. The equivalent bonding layer is introduced by partitioning the original composite, with its thickness allocated from the adjacent steel and aluminum regions rather than added to the overall thickness. Consequently, the bilayer and three-layer FE models retain identical external dimensions, ensuring a consistent basis for comparison.

**Figure 7:**
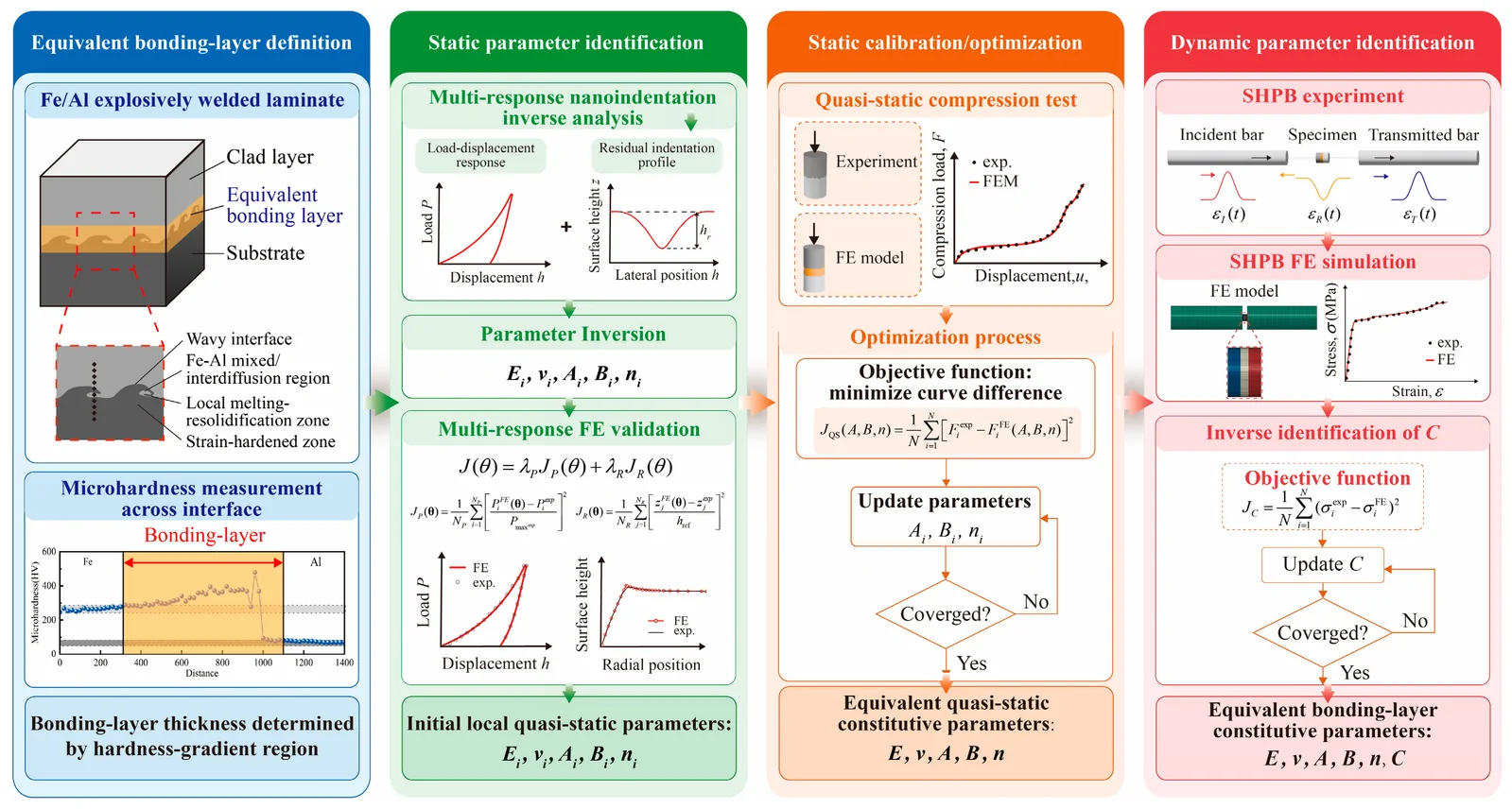
Procedure for identifying the constitutive parameters of the equivalent bonding layer.

The procedure used to identify the constitutive parameters of the equivalent bonding layer is summarized in Figure 7. The microstructural observations, microhardness measurements, nanoindentation results, quasi-static compression responses, and SHPB data reported in this study were obtained from experiments conducted by the authors on the investigated Fe/Al composite and its constituent materials. The cited studies provide methodological support for the constitutive parameter-identification procedures rather than the experimental response data used to calibrate the present model. The Johnson–Cook parameters of the steel clad and aluminum substrate were independently determined from quasi-static compression tests performed using an electronic universal testing machine (D2-50L, Changchun Research Institute for Mechanical Science Co., Ltd., Changchun, China) and SHPB tests. The strain-hardening parameters *A*, *B*, and *n* were obtained from the quasi-static responses, while the strain-rate sensitivity coefficient *C* was identified from the dynamic measurements. The SHPB loading conditions were selected based on the estimated strain rates in the roller–workpiece contact region, with reference to previous cold-beating studies [42]. These constituent material properties were held fixed during the subsequent identification of the equivalent bonding layer.

Nanoindentation tests were conducted at different positions across the bonding region using a Nano Indenter G200 (Agilent Technologies, Santa Clara, CA, USA) to obtain local load–depth responses and residual indentation profiles. Both measurements were incorporated into the inverse FE analysis to provide complementary constraints on the local elastoplastic parameters [43,44,45,46,47]. Equal weighting coefficients (*λ*_P_ = *λ*_R_ = 0.5) were assigned to the normalized discrepancies in the load–depth response and residual indentation profile. The resulting parameter estimates were subsequently used to initialize a genetic algorithm for optimizing the equivalent-layer parameters *A*, *B*, and *n* by minimizing the discrepancy between the experimental and simulated quasi-static compression responses. The effective Young’s modulus was determined through inverse analysis, while Poisson’s ratio was assumed to be 0.25 based on reported properties of Fe–Al intermetallic phases [48]. The combined use of indentation and quasi-static compression responses provides additional constraints on the inverse identification, thereby reducing parameter ambiguity without implying strict mathematical uniqueness. The corresponding experimental and FE comparisons are provided in Supplementary Figures S1 and S3 for nanoindentation and quasi-static compression, respectively.

A local sensitivity analysis was performed by independently varying the equivalent bonding-layer strain-hardening parameters *A*, *B*, and *n* by ±10% and ±20% relative to their identified values, while keeping the remaining parameters unchanged. The predicted quasi-static compression response exhibits the greatest sensitivity to variations in *B*, followed by *A* and *n* (Figure S4).

The strain-rate sensitivity of the equivalent bonding layer was identified using SHPB tests on intact composite specimens with an SHPB apparatus (LWKJ-HPKS-Y20, Luoyang Liwei Technology Co., Ltd., Luoyang, China) [49,50]. Cylindrical specimens with a diameter of 5 mm and a height of 4 mm were prepared by wire electrical discharge machining, grinding, and polishing. The original bonded interface was preserved, with the loading direction perpendicular to the interface and nominal steel and aluminum thicknesses of 2 mm each. In the corresponding three-layer FE model, the steel clad, equivalent bonding layer, and aluminum substrate were assigned thicknesses of 1.6, 0.8, and 1.6 mm, respectively. The tests covered nominal strain rates of approximately 1.2 × 10^3^ to 4.2 × 10^3^ s^−1^, as illustrated in Figure S2. With the quasi-static parameters held fixed, the coefficient *C* was identified by minimizing the discrepancy between the experimental and simulated SHPB responses using a least-squares-based inverse FE procedure.

The identified parameters describe the effective elastic, plastic, and strain-rate-dependent response of the mechanically affected bonding region rather than the intrinsic properties of individual Fe–Al phases. The effective density of the equivalent bonding layer is estimated using the rule of mixtures, with equal nominal volume fractions of steel and aluminum, yielding 5275 kg/m^3^. The constitutive equations and material parameters used for the three material regions are given in Table 2.

### 2.4. Mesh Discretization and Contact Definition

**Figure 8:**
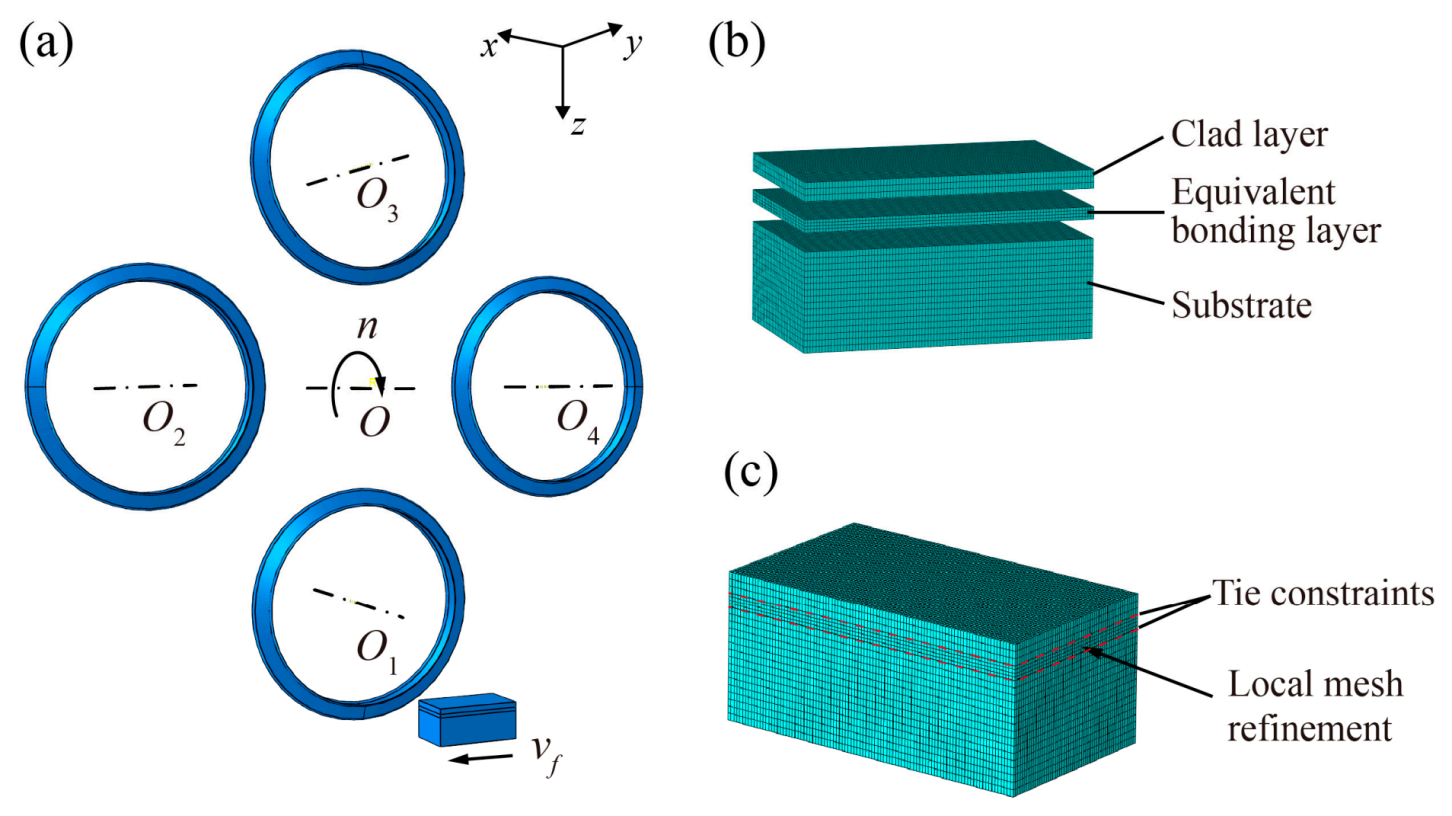
Mesh discretization and interlayer connection scheme of the cold roll-beating FE model: (**a**) overall FE model; (**b**) three-layer workpiece representation; and (**c**) locally refined mesh and tie constraints at the layer boundaries.

The three-dimensional FE model is generated using an Abaqus/Python-based 2020 parametric procedure and solved using the explicit dynamic solver Abaqus/Explicit 2020 (Dassault Systèmes, Vélizy-Villacoublay, France). The roll-beating roller is modeled as an analytical rigid body, as shown in Figure 8a, because its elastic deformation is assumed to be negligible relative to the plastic deformation of the workpiece. This treatment reduces the computational cost of the explicit dynamic analysis without materially affecting the deformation response of the workpiece.

The workpiece is discretized using structured eight-node linear hexahedral elements with reduced integration (C3D8R). Local mesh refinement is applied in the forming zone and around the equivalent bonding layer to capture the high stress gradients and localized plastic deformation in these regions, with a minimum element size of 0.2 mm. A coarser mesh is used in the aluminum substrate away from the contact region, where deformation gradients are relatively small, to reduce unnecessary computational cost.

**Figure 9:**
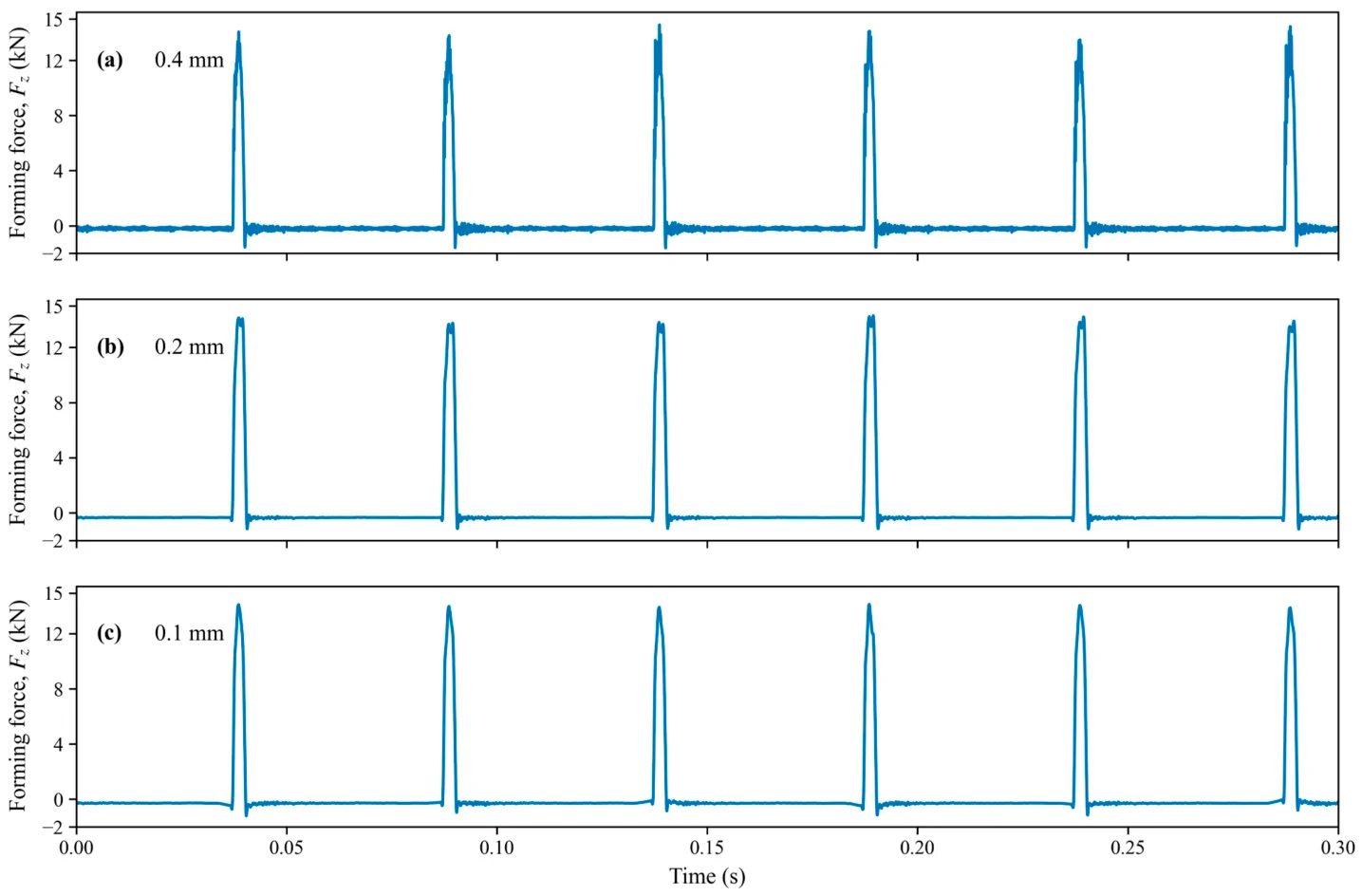
Forming-force histories obtained using different bonding-layer mesh sizes: (**a**) 0.40 mm; (**b**) 0.20 mm; and (**c**) 0.10 mm.

To assess the influence of mesh resolution on the predicted forming response, a mesh sensitivity analysis was conducted using bonding-layer element sizes of 0.40, 0.20, and 0.10 mm, corresponding to two, four, and eight elements through the 0.8 mm equivalent bonding layer, respectively. Figure 9 compares the forming-force histories obtained using the three mesh configurations. The mean peak forces over six successive roller engagements were 14.082, 14.021, and 14.000 kN, respectively, with relative deviations of 0.59% and 0.15% for the 0.40 and 0.20 mm meshes compared with the finest mesh. The coefficient of variation of the periodic peak forces also decreased from 2.87% to 0.72% with mesh refinement. These results indicate that further refinement from 0.20 to 0.10 mm has a limited influence on the predicted peak forming force under the investigated conditions. Accordingly, the 0.20 mm mesh was retained to balance computational efficiency and spatial resolution.

Each material domain is assigned its corresponding elastic properties and strain-rate-dependent J–C plasticity parameters, as summarized in Table 2. The boundaries adjoining the equivalent bonding layer are effective material-partition boundaries rather than direct representations of the actual wavy Fe/Al interface. Tie constraints are imposed at the layer boundaries to enforce displacement continuity under the perfect-bonding assumption, as also adopted in previous FE studies of metallic laminated sheets [52]. Accordingly, the model is intended to predict the macroscopic forming response under the complete-bonding assumption rather than local interfacial damage or failure. Roller–workpiece contact is described using a surface-to-surface formulation, with hard contact in the normal direction and Coulomb friction in the tangential direction. A friction coefficient of *μ* = 0.2 is adopted [51]. A sensitivity analysis was also performed by varying the friction coefficient from 0.1 to 0.3 in increments of 0.1, using *μ* = 0.2 as the baseline value. The selected range represents symmetric perturbations around the baseline and allows the influence of contact friction on the predicted forming force and final profile to be evaluated.

### 2.5. Boundary Conditions and Solution Procedure

**Figure 10:**
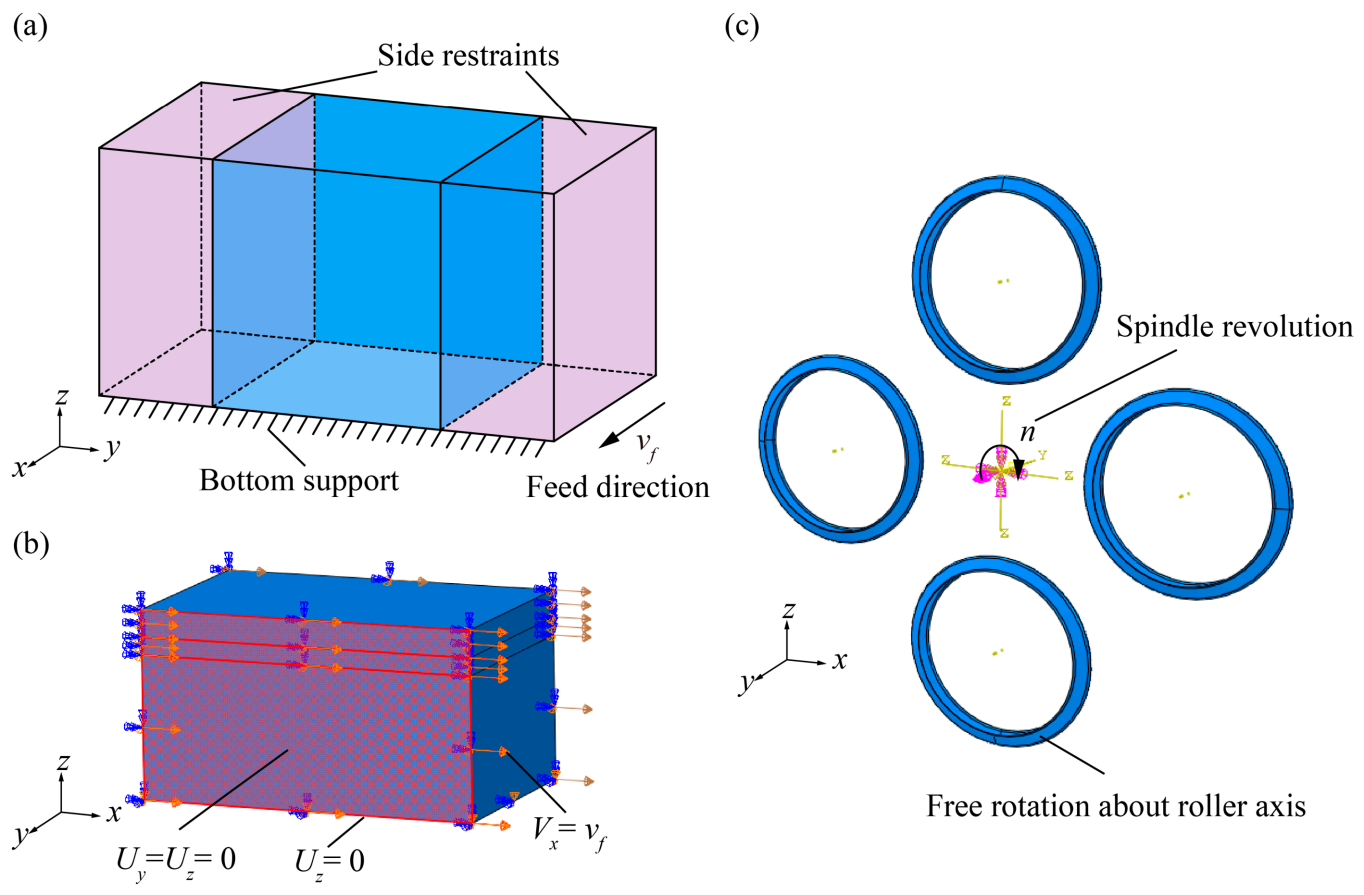
Boundary conditions and prescribed motions of the cold roll-beating FE model: (**a**) schematic of workpiece constraints and feed motion; (**b**) implemented boundary conditions; and (**c**) spindle revolution and passive roller self-rotation.

The boundary conditions and prescribed motions of the finite element model are illustrated in Figure 10. Because only a local segment of the workpiece is modeled, appropriate constraints are imposed to represent the confinement provided by the surrounding material. Translational degrees of freedom in the *y*- and *z*-directions are constrained on the two lateral surfaces, while a constant feed speed *v_f_* is prescribed in the *x*-direction to reproduce the translational motion of the workpiece. The bottom surface is additionally constrained against translation in the *z*-direction to represent support from the fixture, as shown in Figure 10a,b.

Each roller is modeled as an analytical rigid body, with its motion prescribed through the corresponding rigid-body reference point. According to the cold roll-beating kinematics, the roller center revolves periodically about the spindle center *O* along a prescribed circular trajectory. For a spindle speed *n*, the corresponding angular velocity is given by ω = 2πn/60. The initial roller–workpiece position is determined by the nominal roll-beating depth *d*. Rotation about the roller axis is left unconstrained so that passive self-rotation can develop under frictional contact with the workpiece, whereas the remaining degrees of freedom are prescribed according to the roller installation geometry and orbital motion, as illustrated in Figure 10c.

A single roller undergoes a relatively long non-contact interval during each spindle revolution, resulting in considerable computational time being spent on mechanically inactive motion. To improve computational efficiency, a four-roller accelerated-loading scheme is adopted. Four identical rollers are equally spaced around the spindle circumference, while the spindle speed is kept identical to that used in the experiments. The numerical feed speed is correspondingly increased to four times the experimental value. Because the number of effective engagements per spindle revolution increases from one to four, the spacing between successive engagement positions along the feed direction remains unchanged. The engagement spacing is given by

(4) $$s=\frac{v_{f}}{N_{r}n}$$

where *s* is the spacing between successive effective engagements and *N_r_* is the number of effective engagements per spindle revolution.

For the experimental single-roller configuration, *N_r_* = 1, whereas *N_r_* = 4 for the accelerated numerical model. Increasing the numerical feed speed by the same factor preserves the nominal feed displacement per effective engagement and the spatial sequence of successive roller–workpiece contacts. With the spindle speed, roller geometry, orbital radius, and nominal roll-beating depth unchanged, this scheme reduces the non-contact duration while maintaining the spatial engagement relationship governing incremental tooth-profile formation. The corresponding time interval between successive engagements is reduced to one quarter of that in the single-roller configuration. A similar four-roller configuration was adopted by Li et al. [30] in finite element simulations of cold roll-beating forming of homogeneous steel.

Owing to the periodic contact, high local strain rates, and nonlinear material response associated with cold roll-beating, the simulations are performed using the explicit dynamic solver in Abaqus/Explicit. The unloading and subsequent dynamic response between successive roller engagements are retained in the explicit solution. The adopted Johnson–Cook constitutive model accounts for strain hardening and strain-rate-dependent plasticity without introducing an additional time-dependent stress-relaxation law. The total analysis duration is determined from the roller–workpiece relative motion and feed speed, as described in Section 2.2, to ensure that the complete forming process is included. To maintain consistent temporal resolution of the forming-force history and local deformation response under different spindle speeds, the output interval is linked to the effective engagement frequency. If *N_r_* effective engagements occur at equal time intervals during each spindle revolution, the time interval *t_i_* between two successive engagements is given by

(5) $$t_{i}=\frac{60}{N_{r}n}$$

where *t_i_* denotes the time interval between successive effective engagements, and *N_r_* is the number of effective engagements per spindle revolution.

When *N_s_* output points are assigned to each effective engagement interval, the corresponding output time interval Δ*t_out_* for the field and history variables is expressed as

(6) $$\Delta t_{\mathrm{out}}=\frac{t_{i}}{N_{s}}$$

The corresponding output frequency is then given by

(7) $$f_{\mathrm{out}}=\frac{1}{\Delta t_{\mathrm{out}}}=\frac{N_{r}N_{s}n}{60}$$

This setting ensures a comparable number of output points within each effective engagement interval across the investigated process conditions, allowing the forming-force response and internal deformation fields to be evaluated at a consistent temporal resolution.

To improve computational efficiency, a fixed mass-scaling factor of 1000 was adopted in the explicit analysis. Mass scaling increases the stable time increment by increasing the effective mass of the scaled elements, thereby reducing the computational cost. However, excessive mass scaling may introduce additional inertial effects and influence the predicted forming response. Therefore, the numerical energy response was examined to assess the contribution of kinetic and artificial strain energies during the simulation.

**Figure 11:**
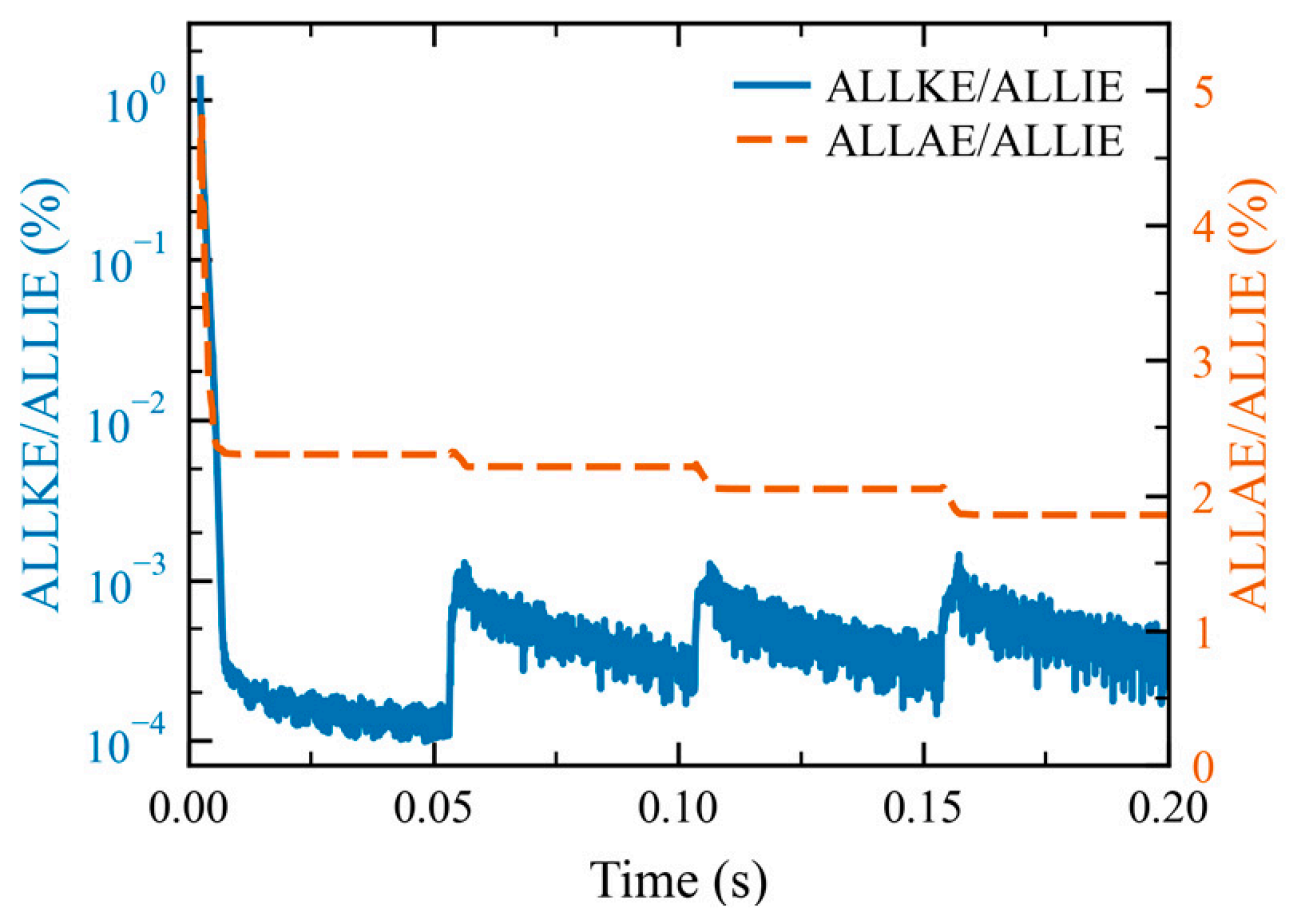
Evolution of the kinetic-to-internal and artificial-to-internal energy ratios during the representative short-duration simulation with a mass-scaling factor of 1000.

Figure 11 presents the evolution of the kinetic-to-internal energy ratio (ALLKE/ALLIE) and the artificial-to-internal energy ratio (ALLAE/ALLIE) obtained from a representative 0.2 s simulation with a mass-scaling factor of 1000. Following the initial roller–workpiece contact, the kinetic-to-internal energy ratio decreased rapidly, with transient increases during subsequent roller engagements. Within the evaluated interval (ALLIE ≥ 0.1 J), the maximum kinetic-to-internal energy ratio was approximately 1.35%. Meanwhile, the artificial-to-internal energy ratio showed a stepwise decreasing trend, reaching approximately 1.86% at the end of the analysis. These results indicate that the kinetic and artificial strain energy contributions remained relatively small during the evaluated interval, supporting the numerical energy assessment of the explicit simulation.

### 2.6. Automated Workflow for Parametric Model Generation

**Figure 12:**
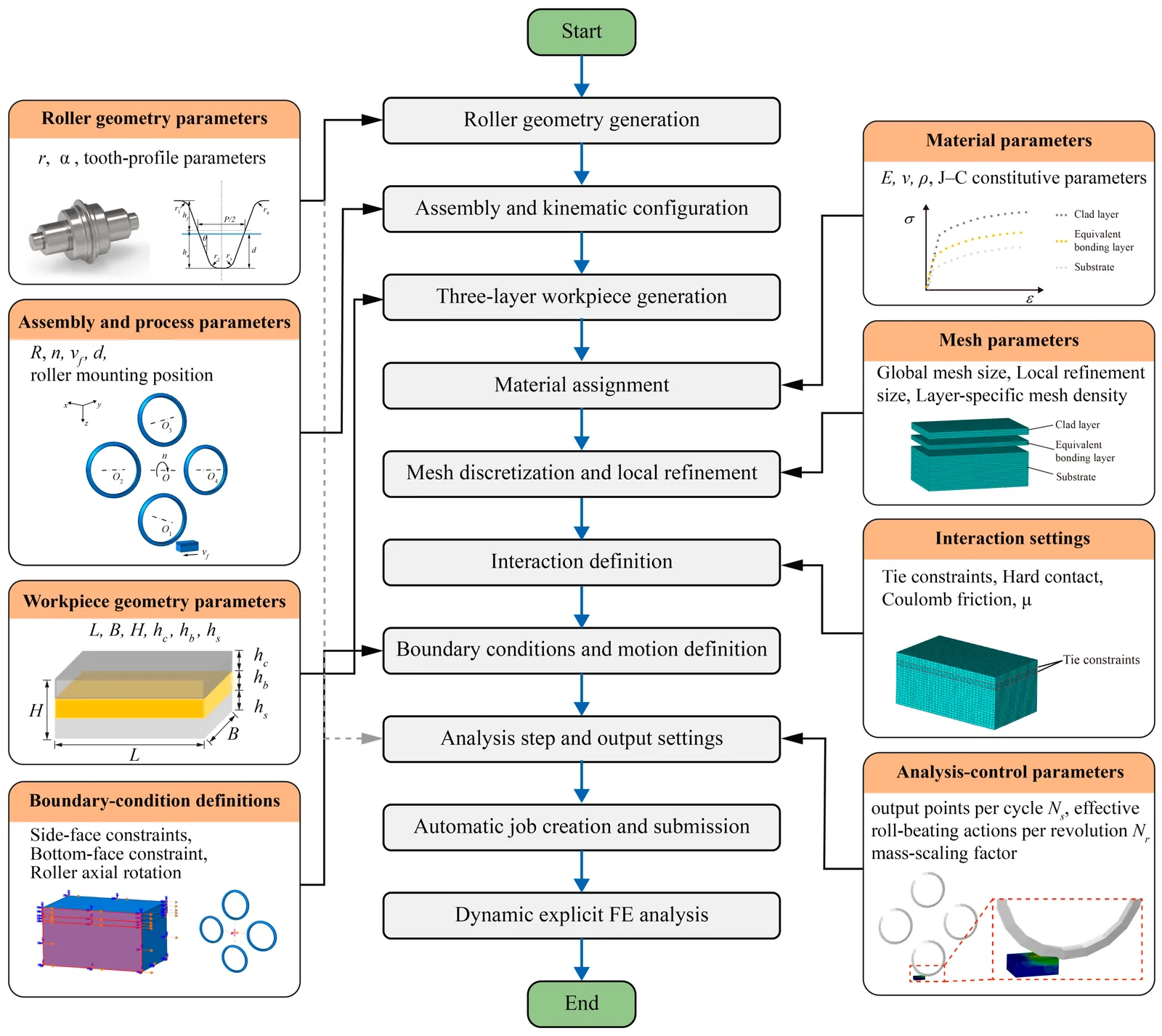
Parameter-driven workflow for automated generation and analysis of cold roll-beating finite element models.

A parameter-driven modeling framework was developed in Abaqus/Python to generate cold roll-beating FE models for different process conditions and layered configurations. The framework integrates roller geometry, process kinematics, layered workpiece geometry, material properties, mesh discretization, contact definitions, boundary conditions, and analysis-control parameters. These modules are executed sequentially according to the prescribed modeling procedure to construct complete FE models from different input parameter sets. The overall workflow is shown in Figure 12.

For each prescribed input set, the program generates the roller and layered workpiece, partitions the material domains, assigns the corresponding constitutive properties, creates the mesh, and defines the contact and boundary conditions. The workpiece dimensions and the thicknesses of the steel clad, equivalent bonding layer, and aluminum substrate remain parametrically linked throughout model construction. This procedure reduces repeated manual operations and improves model consistency across different simulation conditions.

The geometric and process parameters are further used to determine the analysis-control settings. The analysis-step duration is calculated from the roller profile radius *r*, orbital radius *R*, nominal roll-beating depth *d*, workpiece length *L*, and feed speed *v_f_*, while the output interval is determined by the spindle speed *n*, number of effective engagements per revolution *N_r_*, and prescribed number of output points per engagement interval *N_s_*. The analysis-control module also specifies the fixed mass-scaling factor of 1000, as described in Section 2.5. With these parameters specified, the analysis job is generated and submitted to Abaqus/Explicit for solution.

**Figure 13:**
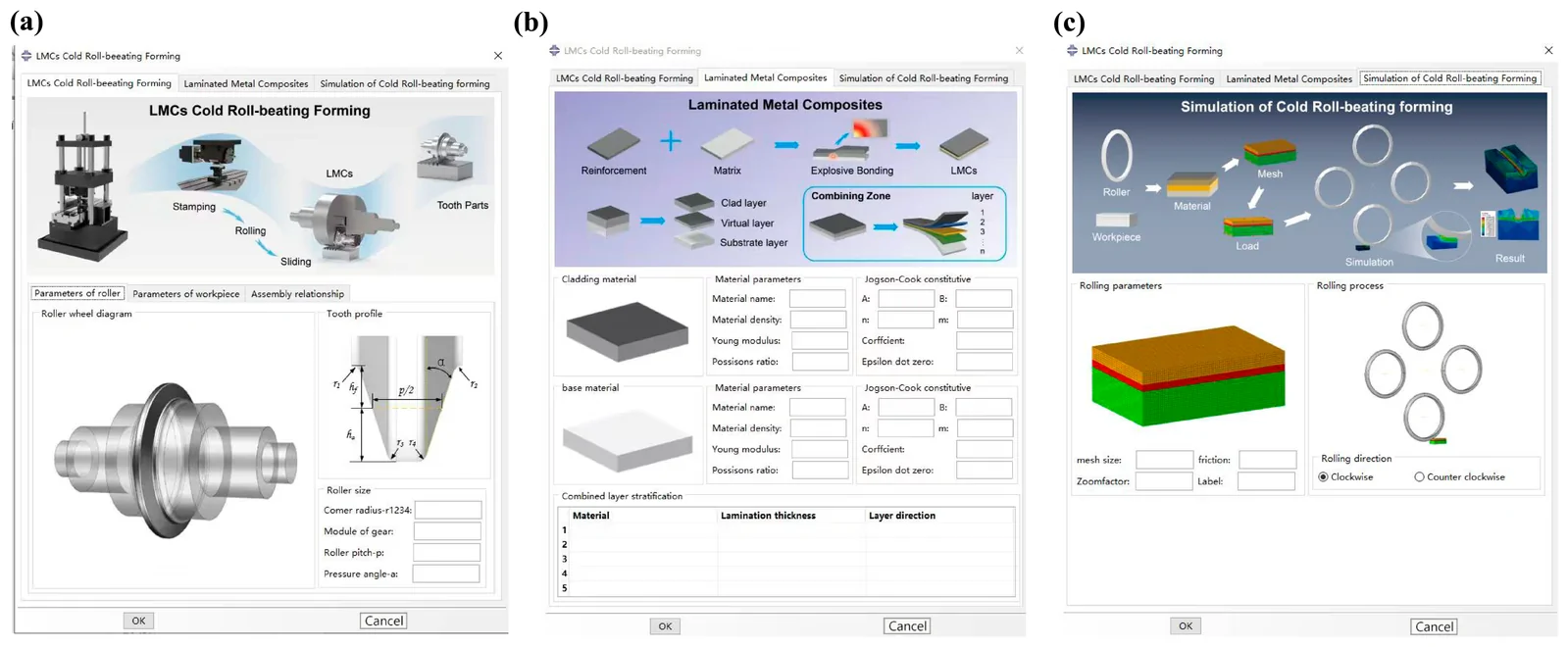
Graphical user interface of the developed parametric modeling framework for cold roll-beating: (**a**) roller geometry parameterization; (**b**) layered composite material and constitutive parameter definition; and (**c**) finite element analysis settings.

A graphical user interface (GUI) was developed for parameter input and model execution, as shown in Figure 13. The interface includes separate modules for roller geometry, process kinematics, layered-workpiece geometry, material properties, and FE analysis settings, as shown in Figure 13a–c. Data entered through these modules are transferred directly to the corresponding model-generation and analysis-control procedures, allowing FE models with different layered configurations and process conditions to be constructed using consistent material, mesh, contact, and boundary-condition definitions. The GUI is used to support the parametric simulations, process-parameter studies, and experimental validation presented in the following sections.

## 3. Experimental Procedure and Model Validation

### 3.1. Experimental Materials and Setup

**Figure 14:**
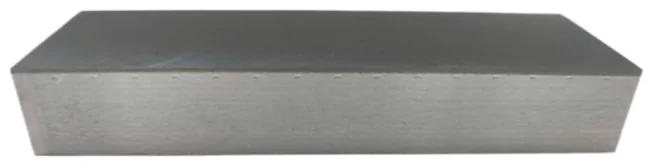
Explosively bonded Fe/Al layered-composite specimen used in the cold roll-beating experiments.

The cold roll-beating experiments were conducted using an explosively bonded Fe/Al layered composite consisting of a steel clad, an aluminum substrate, and an intervening metallurgically bonded region. The specimens measured 130 mm × 40 mm × 20 mm, with nominal steel-clad and aluminum-substrate thicknesses of 1.5 and 18.5 mm, respectively. These values refer to the nominal thicknesses of the original composite, whereas the corresponding material-domain thicknesses in the three-layer FE model are defined using the equivalent-layer procedure described in Section 2.3. The specimen configuration is shown in Figure 14, and the chemical compositions of the steel clad and aluminum substrate are listed in Table 3.

**Figure 15:**
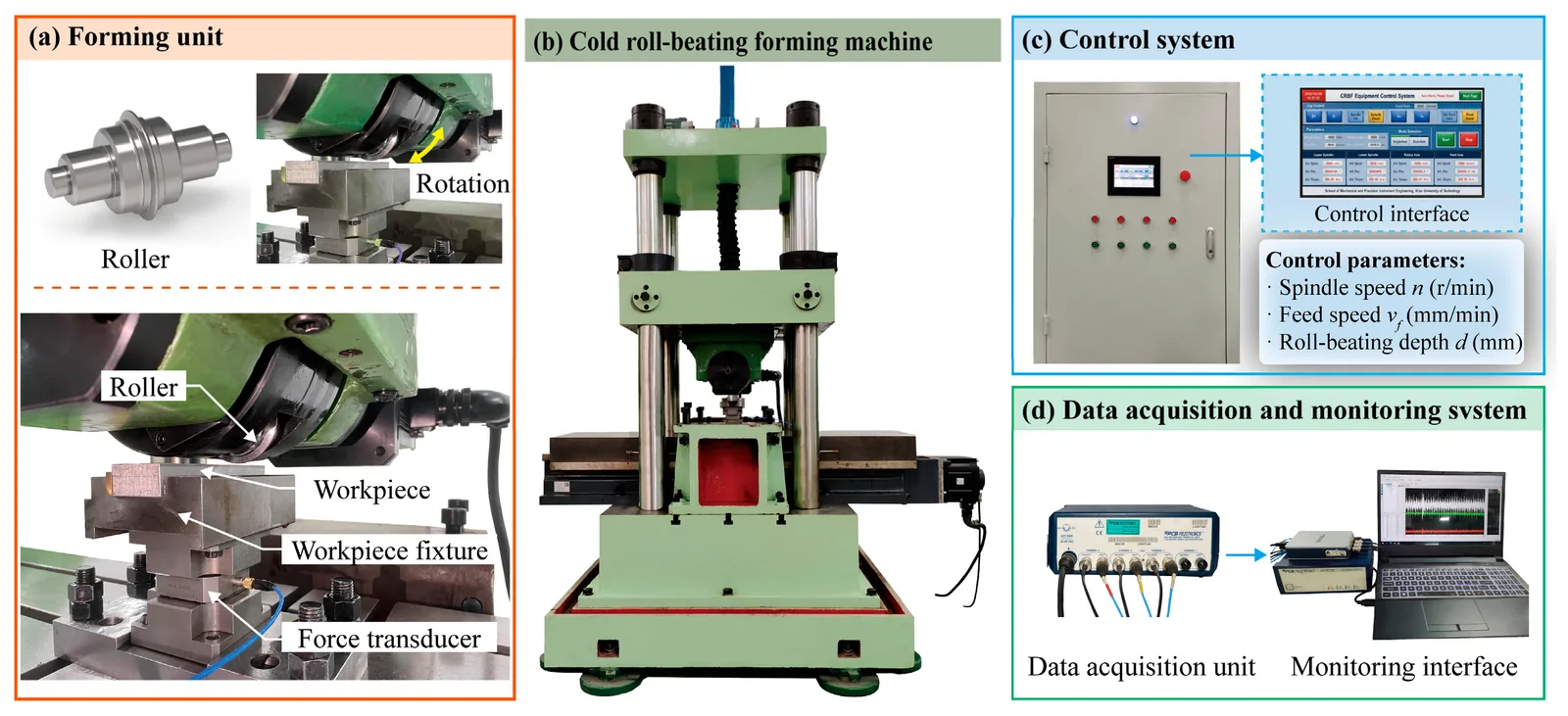
Experimental apparatus and force-measurement system for cold roll-beating of the Fe/Al layered composite: (**a**) roll-beating unit; (**b**) experimental machine; (**c**) control system; and (**d**) force-data acquisition and monitoring system.

The experiments were carried out on a custom-built cold roll-beating test rig, as shown in Figure 15. The machine uses a three-beam, four-column frame with the roll-beating unit mounted at the center. The spindle speed and workpiece feed speed are controlled independently, while the nominal roll-beating depth is adjusted through the relative position between the spindle and workpiece, as shown in Figure 15a–c.

The workpiece is clamped to the worktable using a dedicated fixture, beneath which a PCB261A03 triaxial force transducer (PCB Piezotronics, Depew, NY, USA) with a measurement range of 40 kN is installed. The workpiece, fixture, and force transducer move together with the worktable, and the three-component force signals are recorded continuously at 50 kHz, as shown in Figure 15d. The stable-stage force data are used for subsequent forming-force evaluation and FE model validation.

### 3.2. Cold Roll-Beating Experiments and Measurements

Spindle speed *n* and feed speed *v_f_* were selected as the primary process variables, with the test conditions listed in Table 4. The spindle speed ranged from 400 to 2400 r/min and the feed speed from 100 to 225 mm/min. The nominal roll-beating depth was fixed at *d* = 2.37 mm for all tests according to the target tooth geometry. Each condition was repeated three times under the same lubrication conditions.

**Figure 16:**
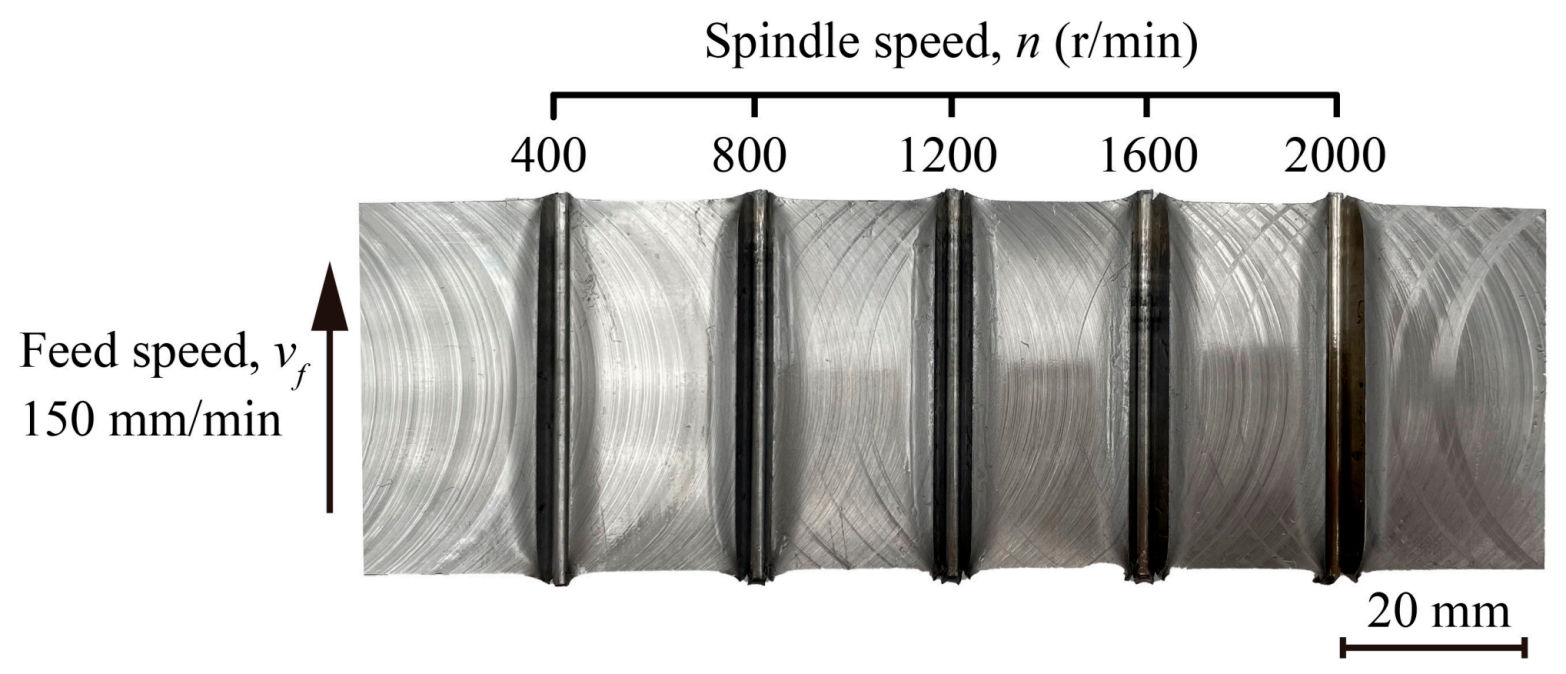
Representative grooves formed in the Fe/Al layered composite at different spindle speeds.

**Figure 17:**
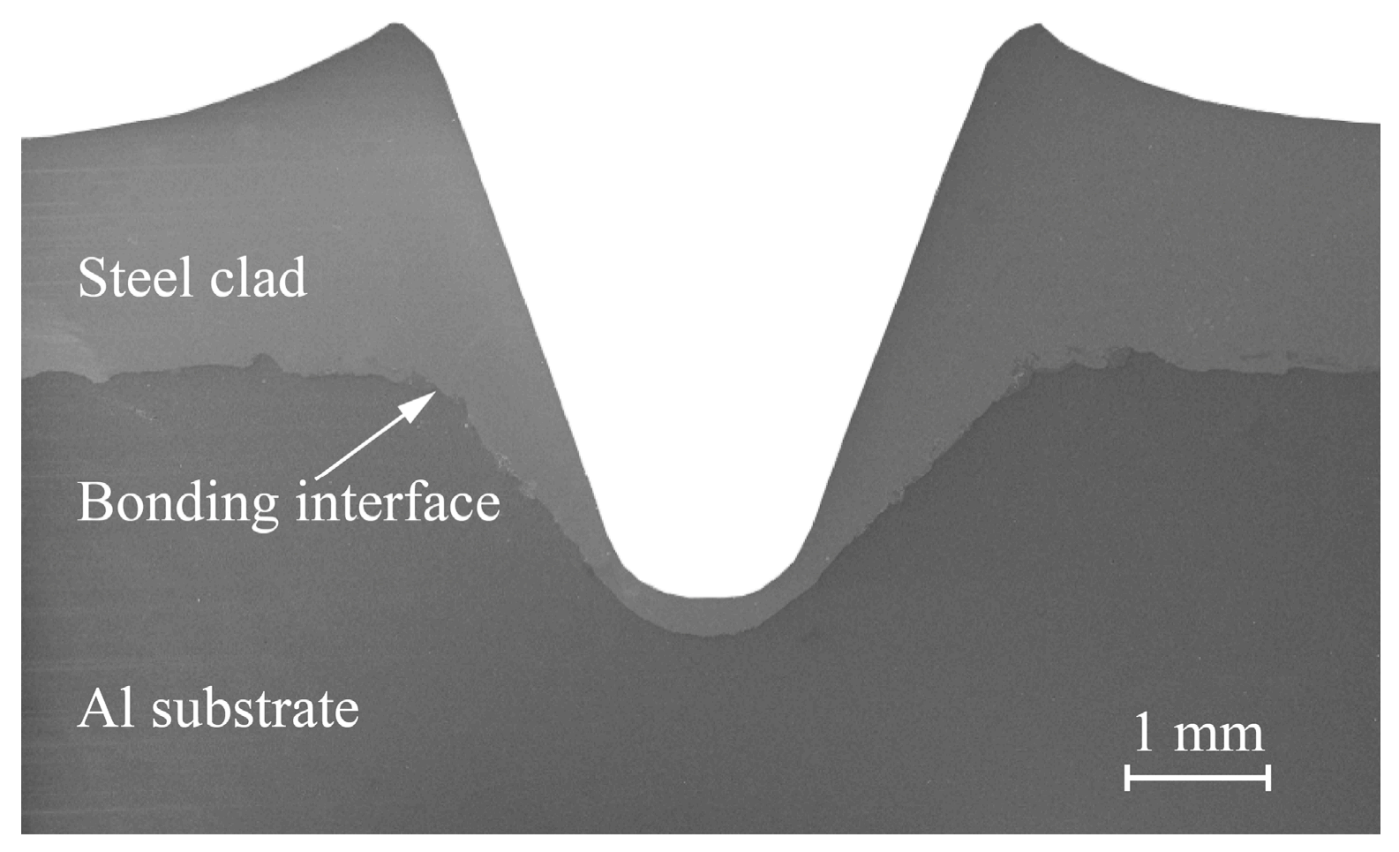
SEM cross-sectional morphology of a representative groove formed in the explosively bonded Fe/Al layered composite.

Figure 16 shows representative formed grooves obtained at different spindle speeds with *v_f_* = 150 mm/min and *d* = 2.37 mm. The cross-sectional morphology of a representative formed groove was further examined by SEM, as shown in Figure 17. The Fe/Al bonding interface remains visually continuous along the groove root and adjacent flanks, with no apparent interfacial opening or macroscopic delamination in the examined section. This observation is consistent with the perfect-bonding assumption adopted in the FE model.

**Figure 18:**
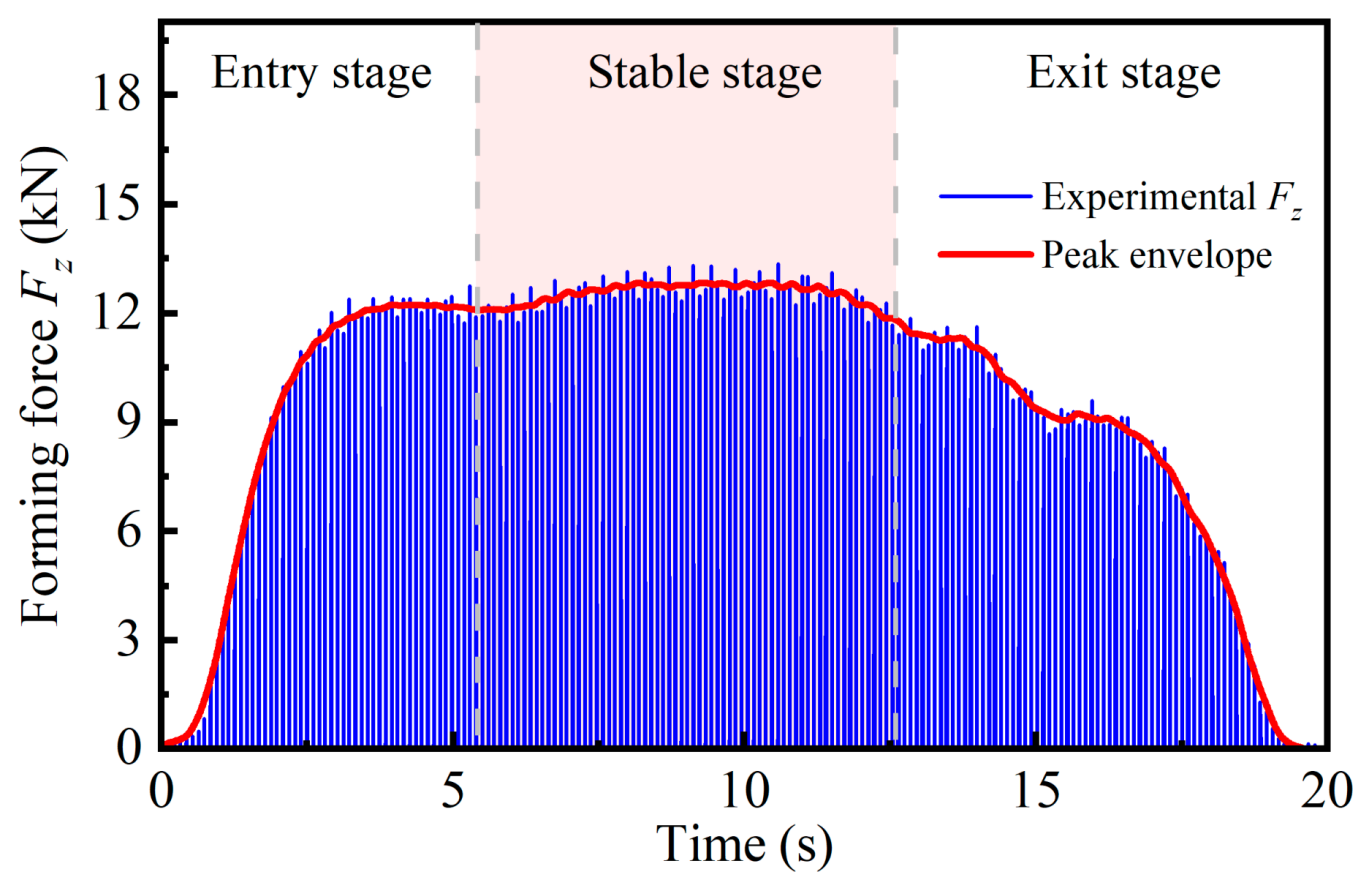
Representative experimental *F_z_* response and peak-force envelope during cold roll-beating.

During testing, the workpiece, fixture, and force-measurement assembly translated together with the worktable in the *x*-direction. The normal forming force *F_z_*, acting in the *z*-direction perpendicular to the initial workpiece surface, was selected as the primary load metric. A representative force–time history obtained at *n* = 400 r/min, *v_f_* = 150 mm/min, and *d* = 2.37 mm is shown in Figure 18. The response can be divided into three stages: engagement, stable forming, and disengagement. During engagement, the contact force increases as the roller enters the forming region. The stable forming stage is characterized by a repeatable periodic force response, whereas the disengagement stage corresponds to the progressive loss of roller–workpiece contact.

To minimize the influence of the transient responses associated with initial engagement and final disengagement, subsequent load evaluation was based on the *F_z_* signal recorded during the stable forming stage. The measured force signal was processed using a fourth-order Butterworth low-pass filter with a cutoff frequency of 2 kHz. Zero-phase filtering was applied to avoid phase distortion, after which the peak *F_z_* value was extracted from each effective engagement within the selected stable-stage interval. These peak values were averaged to obtain the characteristic forming force for each individual test. The characteristic forces obtained from the three independent repetitions were then averaged to determine the representative experimental forming force for each process condition. The standard deviation of the three characteristic forces was also calculated to characterize the variability among independent experiments, separately from the fluctuations of individual engagement peaks within each test.

The formed surface was measured using a VHX-5000 three-dimensional digital microscope (KEYENCE Corporation, Osaka, Japan). A representative cross-section within the stable forming region is selected from each specimen to extract the two-dimensional profile. The experimentally measured profile and the corresponding finite element prediction are then transformed to a common coordinate system, providing the basis for subsequent quantitative evaluation of profile-prediction accuracy.

### 3.3. Experimental Validation of the FE Model

To evaluate the predictive capability of the developed finite element model for cold roll-beating of Fe/Al layered composites, the simulations are performed using the same roller geometry, orbital radius, spindle speed, and nominal roll-beating depth as those used in the experiments. Because the numerical model employs the four-roller accelerated-loading scheme described in Section 2.5, the numerical feed speed is adjusted so that the spacing between successive effective engagements remains identical to that of the experimental single-roller configuration. The experimental and numerical models differ in effective forming length and, consequently, in the duration of the stable forming stage. The complete force–time histories are therefore not compared directly. Model validation is instead carried out within the stable forming region, away from the transient engagement and disengagement stages, using both forming force and final profile. The characteristic forming force is taken as the mean peak *F_z_* over successive effective engagements, while profile accuracy is quantified by the deviation between the measured and predicted profiles.

**Figure 19:**
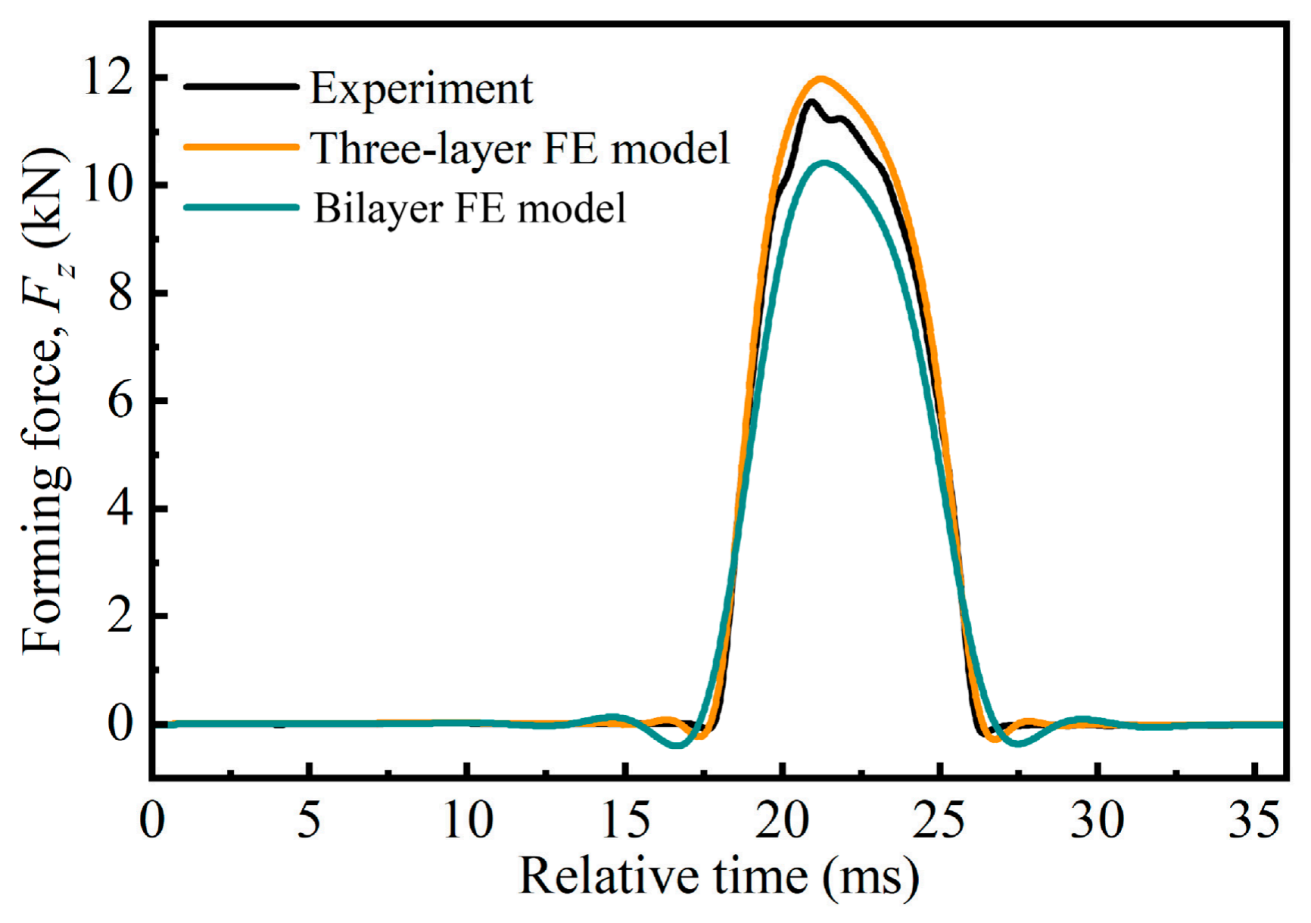
Comparison of experimental and predicted force responses during a representative effective engagement for the three-layer and bilayer FE models (*n* = 1200 r/min, *v_f_* = 150 mm/min, and *d* = 2.37 mm).

Figure 19 compares the force responses during a representative effective engagement in the stable forming stage obtained from the experiment, the three-layer FE model, and the simplified bilayer model. The simplified bilayer model represents the Fe/Al boundary as an idealized, zero-thickness, perfectly bonded interface, whereas the three-layer model explicitly incorporates a finite-thickness equivalent bonding region with independently identified effective constitutive properties. This comparison is therefore intended to assess the contribution of the bonding region to the predicted macroscopic forming response. All three curves show a rapid rise to the peak force followed by gradual unloading, consistent with the local loading characteristics of cold roll-beating. The three-layer model predicts a peak force closer to the experimental result, whereas the bilayer model gives a noticeably lower value.

**Figure 20:**
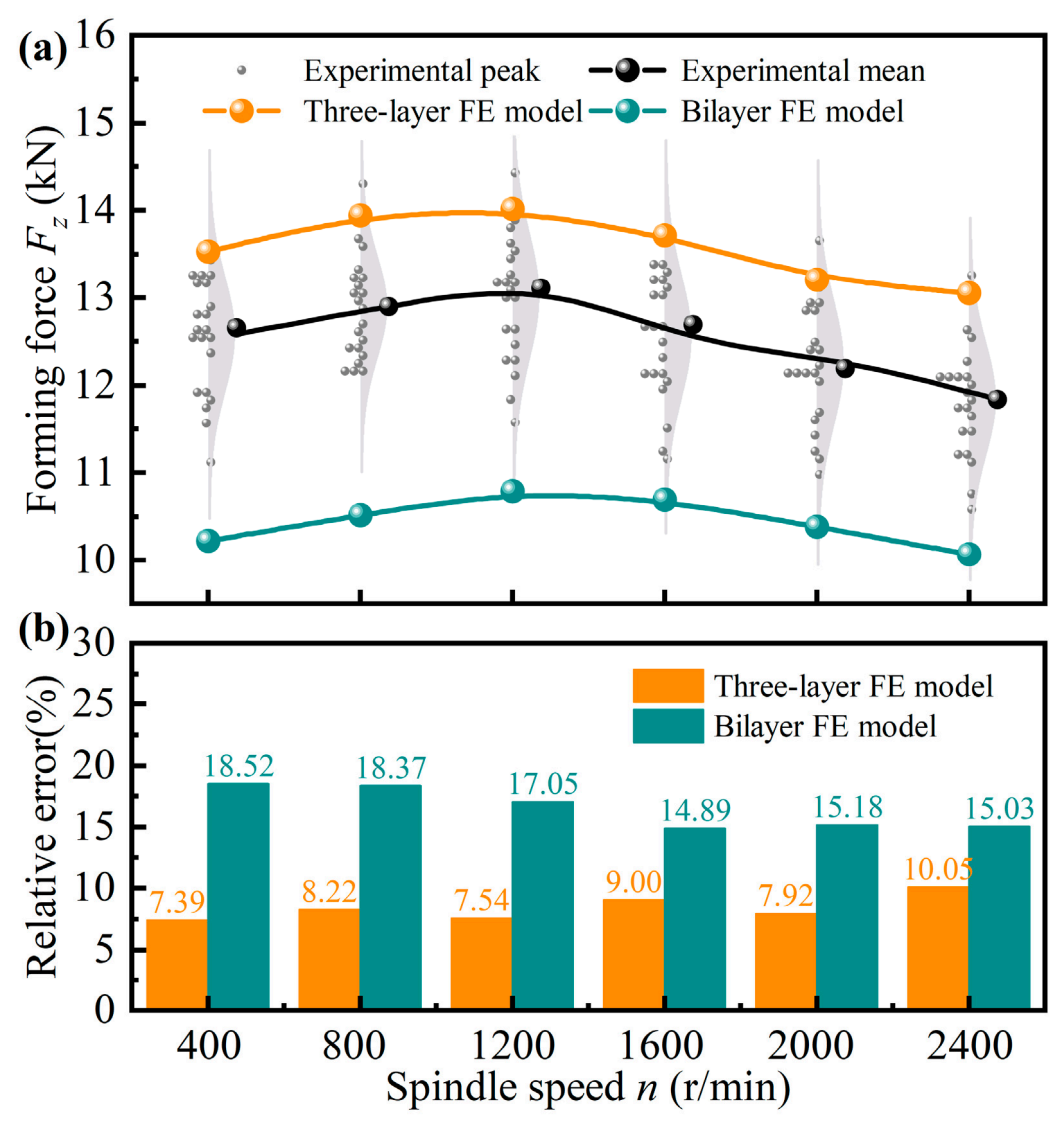
Comparison of experimental and predicted forming-force results at different spindle speeds: (**a**) mean peak forming forces and experimental peak-force distributions; (**b**) absolute relative errors of the FE predictions.

To assess model performance over the investigated spindle-speed range, Figure 20a compares the experimentally measured and numerically predicted mean peak forming forces within the stable forming stage. The gray scatter points and half-violin plots in Figure 20a represent the distributions of peak forming forces extracted from selected effective engagements within the stable forming stages of three independent experiments for each condition. The black curve denotes the representative experimental mean obtained from the three tests, whereas the FE curves represent deterministic predictions. As the spindle speed increases from 400 to 1200 r/min, the experimentally measured mean peak forming force increases from 12.58 to 13.02 kN and subsequently decreases to 11.84 kN at 2400 r/min, exhibiting a non-monotonic dependence on spindle speed. Both FE models reproduce this overall trend, although the three-layer model provides closer agreement with the experimental measurements, whereas the bilayer model consistently underpredicts the forming force.

Figure 20b quantifies the corresponding prediction errors. Across the six spindle-speed conditions, the absolute relative errors of the three-layer model range from 7.39% to 10.05%, with a mean value of 8.35%. The corresponding errors of the bilayer model range from 14.89% to 18.52%, with a mean value of 16.51%. Incorporating the equivalent bonding layer therefore reduces the mean absolute relative error by approximately 49.4%.

**Figure 21:**
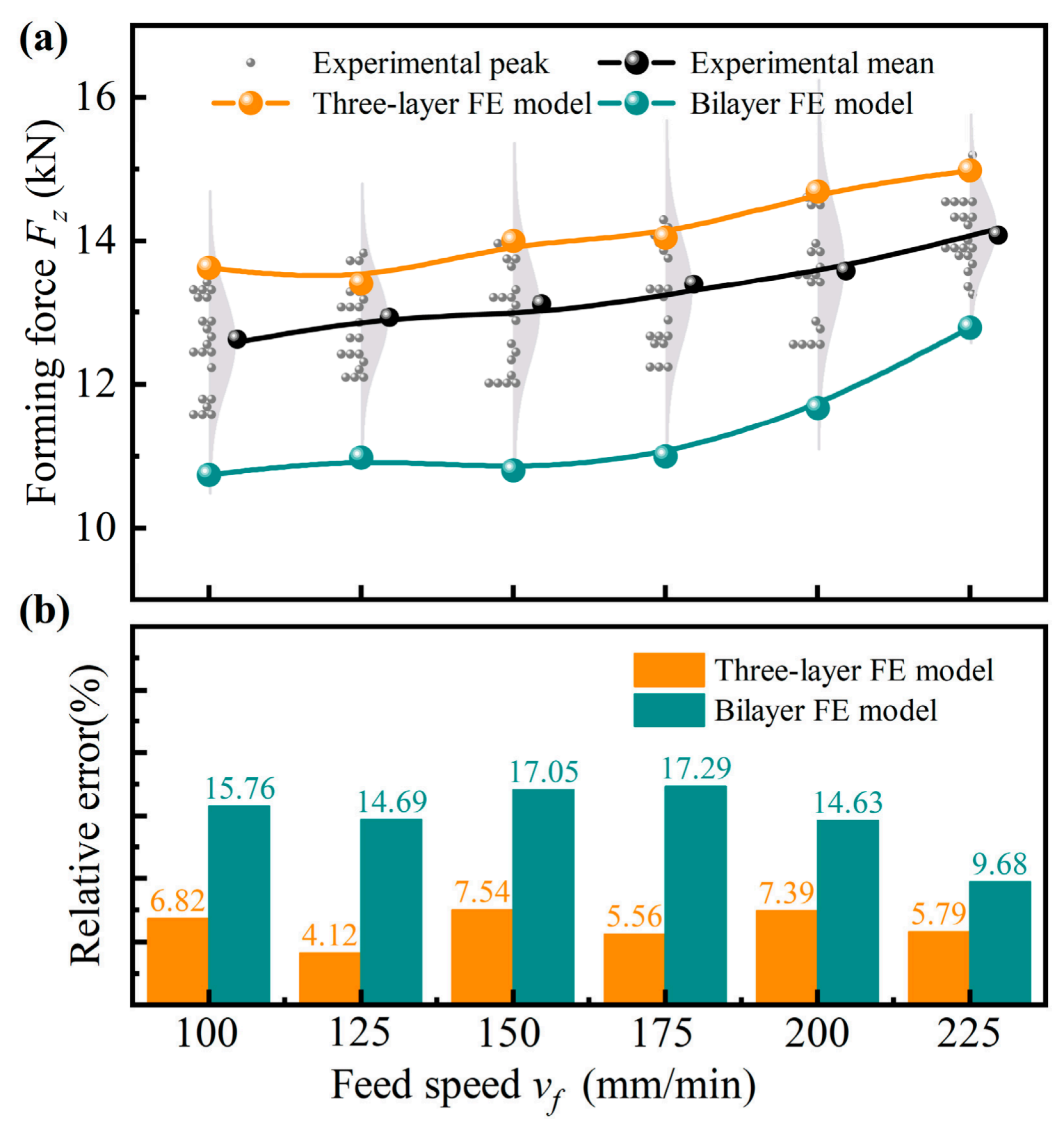
Comparison of experimental and predicted mean peak forming forces and corresponding absolute relative errors at different feed speeds: (**a**) mean peak forming forces and experimental peak-force distributions; (**b**) absolute relative errors of the FE predictions.

The predictive performance of the two models is further evaluated over the investigated feed-speed range, as shown in Figure 21. At a fixed spindle speed of 1200 r/min and a nominal roll-beating depth of 2.37 mm, the experimentally measured mean peak forming force increases from 12.75 to 14.16 kN as the feed speed rises from 100 to 225 mm/min. Both models reproduce the overall trend, but the three-layer predictions remain closer to the experimental measurements, whereas the bilayer model systematically underpredicts the forming force.

As shown in Figure 21b, the absolute relative errors of the three-layer model range from 4.12% to 7.54%, with a mean value of 6.20%. The corresponding errors of the bilayer model range from 9.68% to 17.29%, with a mean value of 14.85%. Incorporating the equivalent bonding layer reduces the mean absolute relative error by approximately 58.2% over the investigated feed-speed range.

Taken together, the spindle-speed and feed-speed validation results show that the three-layer model achieves lower forming-force prediction errors than the bilayer model over the investigated process range. For a more direct quantitative assessment, Table 5 summarizes the experimental mean peak forming forces and their standard deviations from three independent tests, together with the corresponding predictions and absolute relative errors of the three-layer and bilayer FE models under different spindle-speed and feed-speed conditions. The absolute differences between the three-layer FE predictions and the experimental mean forces exceeded the corresponding experimental standard deviations under all investigated conditions.

**Figure 22:**
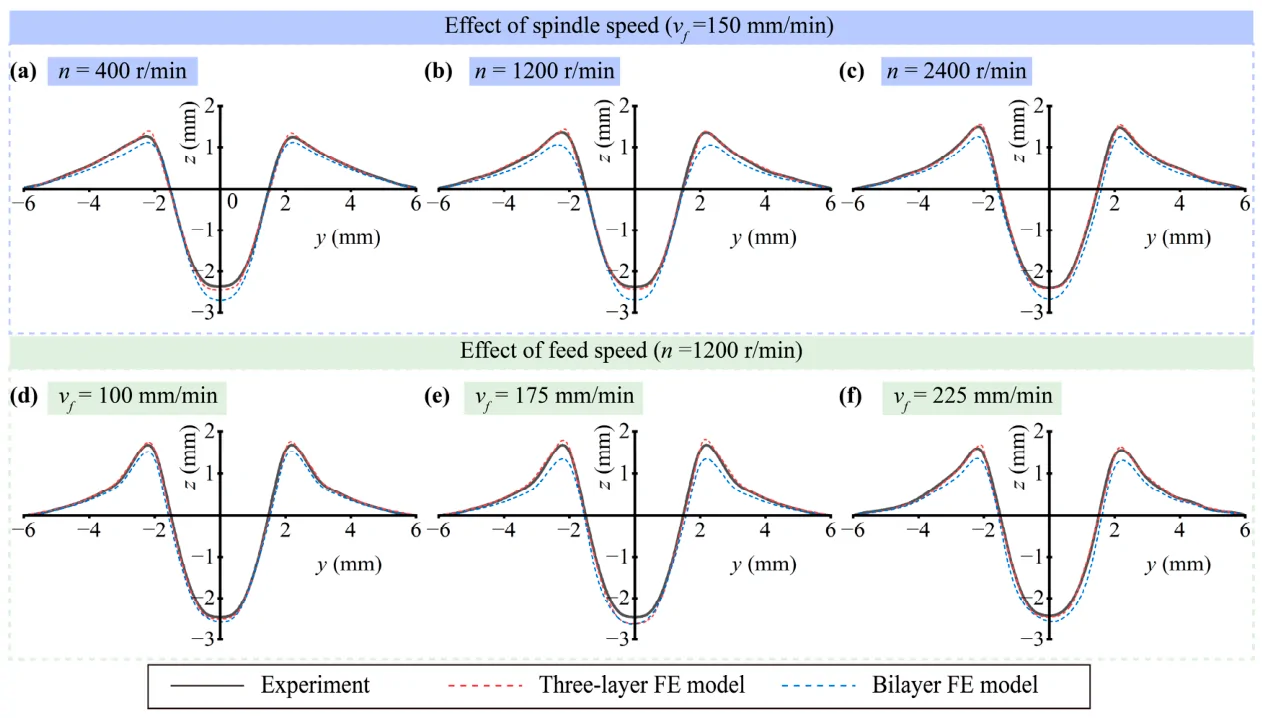
Comparison of experimentally measured and numerically predicted formed profiles under representative process conditions: (**a**–**c**) spindle speeds of 400, 1200, and 2400 r/min at *v_f_* = 150 mm/min; (**d**–**f**) feed speeds of 100, 175, and 225 mm/min at *n* = 1200 r/min.

In addition to forming force, the predictive capability of the model is evaluated in terms of the final formed profile. Representative conditions at low, intermediate, and high spindle speeds and feed speeds are selected for comparison, as shown in Figure 22. Figure 22a–c show the profiles obtained at spindle speeds of 400, 1200, and 2400 r/min, respectively, with *v_f_* = 150 mm/min and *d* = 2.37 mm. Figure 22d–f show the corresponding comparisons at feed speeds of 100, 175, and 225 mm/min, respectively, with *n* = 1200 r/min and *d* = 2.37 mm.

Across the investigated conditions, the three-layer model closely reproduces the tooth-root depth, material pile-up on both sides of the groove, and the geometry of the flank transition regions. By comparison, the bilayer model generally predicts a deeper tooth root, less pronounced material pile-up, and larger local deviations in the flank regions. These comparisons show that the three-layer model provides closer agreement with the measured final profiles under the investigated conditions.

To reduce the subjectivity inherent in qualitative profile comparisons, three quantitative metrics are introduced: the normalized root-mean-square error (NRMSE) of the overall profile, the relative error in tooth-root depth, and the relative error in tooth-crest height.

Because the experimental and numerical profiles differ in sampling locations and point densities, both profiles are first aligned within a common coordinate system. Shape-preserving piecewise cubic Hermite interpolation (PCHIP) is then used to resample both profiles onto a common uniformly spaced horizontal grid [53]. The root-mean-square error (RMSE) is calculated from the resampled data as

(8) $$\mathrm{RMSE}=\sqrt{\frac{1}{N}\sum_{i=1}^{N}{\left(z_{i}^{\mathrm{FE}}-z_{i}^{\exp}\right)}^{2}}$$

where *N* is the number of sampling points on the common coordinate grid, while $z_{i}^{\mathrm{FE}}$ and $z_{i}^{\exp}$ denote the numerically predicted and experimentally measured profile heights, respectively, at the *i*th horizontal position.

To account for differences in profile-height range among the investigated conditions, the RMSE is normalized by the peak-to-valley height of the corresponding experimental profile:

(9) $$\mathrm{NRMSE}=\frac{\mathrm{RMSE}}{z_{\max}^{\exp}-z_{\min}^{\exp}}\times 100\%$$

where $z_{\max}^{\exp}$ and $z_{\min}^{\exp}$ are the maximum and minimum heights of the experimental profile, respectively.

In addition to the overall profile error, the tooth-root depth and tooth-crest height are used to quantify local geometric accuracy. Taking the initial workpiece surface as the height reference, the tooth-root depth is defined as *h*_root_ = |*z*_min_| and its relative error is calculated as

(10) $$\delta_{\mathrm{root}}=\frac{\left|h_{\mathrm{root}}^{\mathrm{FE}}-h_{\mathrm{root}}^{\exp}\right|}{h_{\mathrm{root}}^{\exp}}\times 100\%$$

Because crest features occur on both sides of the formed groove, the mean of the left and right crest heights is used as the characteristic tooth-crest height:

(11) $$h_{\mathrm{crest}}=\frac{h_{\mathrm{crest},L}+h_{\mathrm{crest},R}}{2}$$

The corresponding relative error is

(12) $$\delta_{\mathrm{crest}}=\frac{\left|h_{\mathrm{crest}}^{\mathrm{FE}}-h_{\mathrm{crest}}^{\exp}\right|}{h_{\mathrm{crest}}^{\exp}}\times 100\%$$

The quantitative profile-prediction errors are summarized in Table 6. Across the six representative conditions, the three-layer model yields NRMSE values of 2.74–3.80%, with a mean of 3.27%, whereas the bilayer model gives values of 4.44–5.84%, with a mean of 5.25%. The equivalent-bonding-layer representation therefore reduces the mean NRMSE by approximately 37.7%, and the three-layer model gives a lower overall profile error in all six cases.

The three-layer model also gives mean relative errors of 3.60% for tooth-root depth and 7.25% for tooth-crest height, compared with 9.37% and 13.85%, respectively, for the bilayer model. The three-layer model produces lower tooth-crest height errors in all six cases and lower tooth-root depth errors in five out of the six investigated conditions. The improvement is not uniform for every local geometric feature, however. As shown in Figure 22e, at *v_f_* = 175 mm/min, the bilayer model predicts a tooth-root depth closer to the experimental measurement, with a relative error of 3.26%, compared with 6.85% for the three-layer model. However, the bilayer model underestimates material pile-up in the adjacent crest regions. The three-layer model gives a lower tooth-crest height error of 7.52%, compared with 17.98% for the bilayer model, and a lower overall profile NRMSE of 2.74%, compared with 5.16%. These results indicate that improved overall profile agreement does not necessarily imply improved prediction of every local geometric feature, since the profile NRMSE evaluates geometric deviations across the entire sampled profile rather than a single local feature.

To further evaluate the influence of the effective constitutive properties of the bonding region, two additional control simulations are performed under a representative forming condition. The equivalent bonding layer is assigned the material properties of either the steel clad or the aluminum substrate, while the three-layer geometry and other modeling conditions remain unchanged. The resulting mean peak forming forces and profile NRMSE values are compared with those of the original bilayer and three-layer models, as summarized in Table 7.

As shown in Table 7, the material properties assigned to the equivalent bonding layer influence both the predicted forming force and final profile. Compared with the original three-layer model, the steel-property control model predicts a slightly higher forming force, while the profile NRMSE increases only marginally from 3.80% to 4.20%. In contrast, the aluminum-property control model substantially underpredicts the experimental forming force and exhibits a larger profile deviation. Among the investigated models, the three-layer model with experimentally identified equivalent properties provides the best overall agreement with the experimental results, yielding the lowest force error of 7.54% and profile NRMSE of 3.80%. Although the steel-property control model achieves comparable profile accuracy, its force prediction error increases to 9.91%. These results highlight the importance of evaluating both forming-force and final-profile accuracy when assessing the predictive performance of the equivalent-layer model.

Across the investigated conditions, the three-layer model systematically overpredicts the experimental forming force, whereas the bilayer model systematically underpredicts it. These opposing deviations may be associated with the different representations of the mechanically affected bonding region. The bilayer model neglects its finite-thickness effective response, while the three-layer model approximates the spatially heterogeneous region using homogeneous constitutive properties. The material-property control simulations further confirm that the constitutive properties assigned to the bonding region influence the predicted forming force. However, the remaining discrepancy cannot be attributed exclusively to the homogeneous equivalent-layer representation, as the effects of different modeling assumptions have not been independently isolated. Overall, these results indicate that incorporating the experimentally identified effective properties improves the macroscopic forming-force prediction, although systematic deviations remain.

The effect of the friction coefficient on the predicted forming force and final profile is further evaluated under a representative forming condition. As summarized in Supplementary Table S1, increasing μ from 0.1 to 0.3 raises the predicted mean peak forming force from 13.968 to 14.151 kN, corresponding to a total change of approximately 1.31% relative to the baseline prediction at *μ* = 0.2. The corresponding absolute relative errors are 7.28%, 7.54%, and 8.69%, respectively. The profile NRMSE values are 5.13%, 3.80%, and 9.30%, with the lowest value obtained at *μ* = 0.2. Thus, although the predicted forming force varies only slightly over the investigated friction range, the agreement between the predicted and experimental final profiles shows more noticeable differences. The corresponding forming-force histories and final-profile comparisons are presented in Supplementary Figures S5 and S6, respectively.

Overall, the forming-force and profile comparisons show that the experimentally informed three-layer model provides more accurate predictions than the simplified bilayer model over the investigated process range. The validated three-layer model therefore provides the basis for the subsequent analysis of process-parameter effects and internal deformation behavior.

## 4. Results and Discussion

### 4.1. Effects of Spindle Speed and Feed Speed on Forming Force

**Figure 23:**
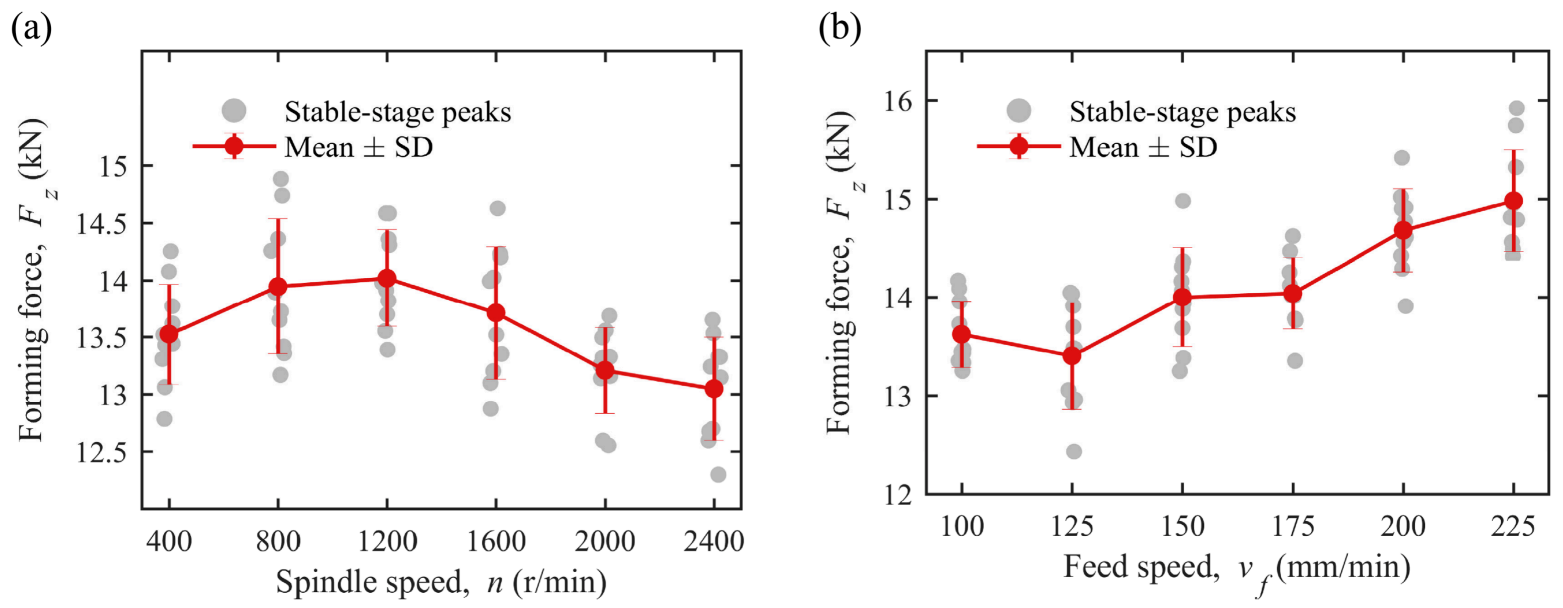
Effects of spindle speed and feed speed on the stable-stage peak forming force: (**a**) spindle speed at *v_f_* = 150 mm/min; (**b**) feed speed at *n* = 1200 r/min.

Based on the validation results, the three-layer model incorporating the equivalent bonding layer is used to investigate the effects of spindle speed *n* and feed speed *v_f_* on the forming force during cold roll-beating of the Fe/Al layered composite. The nominal roll-beating depth is fixed at *d* = 2.37 mm for all cases, and the normal forming force *F_z_* during the stable forming stage is used for comparison. In Figure 23, the gray markers represent the peak force associated with individual effective engagements, while the red symbols and error bars indicate the corresponding mean values and standard deviations, respectively.

Figure 23a shows the effect of spindle speed at a fixed feed speed of *v_f_* = 150 mm/min. As the spindle speed increases from 400 to 1200 r/min, the mean peak forming force increases slightly, whereas a further increase in spindle speed to 2400 r/min leads to a gradual decrease. The resulting dependence on spindle speed is therefore non-monotonic. This non-monotonic response is associated with simultaneous changes in the local loading rate and the deformation history during successive roller engagements. Increasing the spindle speed raises the roller velocity and local loading rate within the contact region. Because the constituent materials exhibit strain-rate-sensitive behavior, the associated increase in plastic strain rate tends to increase the instantaneous flow stress and, consequently, the peak forming force [42]. At a constant feed speed, the spacing between successive engagements decreases with increasing spindle speed. For the single-roller configuration, the engagement spacing is *s* = *v_f_*/*n*; at *v_f_* = 150 mm/min, increasing *n* from 400 to 2400 r/min reduces *s* from 0.375 to 0.0625 mm. The smaller spacing increases the overlap between adjacent deformation regions, so each subsequent engagement acts on material that has undergone more prior deformation. The non-monotonic force response is therefore associated with the combined influence of strain-rate sensitivity and the changing deformation state caused by repeated local loading.

Figure 23b shows the effect of feed speed at *n* = 1200 r/min. As *v_f_* increases from 100 to 225 mm/min, the mean peak forming force rises from 13.62 to 14.98 kN, corresponding to an increase of approximately 10.0%. Over the same range, the theoretical spacing between successive engagements increases from approximately 0.083 to 0.188 mm. At lower feed speeds, the stronger overlap between adjacent deformation regions allows more deformation to accumulate before the subsequent engagement. With increasing feed speed, this overlap is reduced and each engagement acts on material with less prior deformation. Consequently, a larger incremental deformation is imposed during each engagement, leading to the observed increase in peak forming force.

Both spindle speed and feed speed affect the spacing between successive engagements and thus the extent of repeated loading and plastic-deformation accumulation along the feed direction. However, spindle speed also changes the roller velocity and local loading rate, whereas feed speed mainly influences the engagement spacing and deformation overlap. The peak forming force therefore reflects the local resistance during an individual engagement rather than the overall extent of accumulated profile deformation.

### 4.2. Effects of Spindle Speed and Feed Speed on the Formed Profile

**Figure 24:**
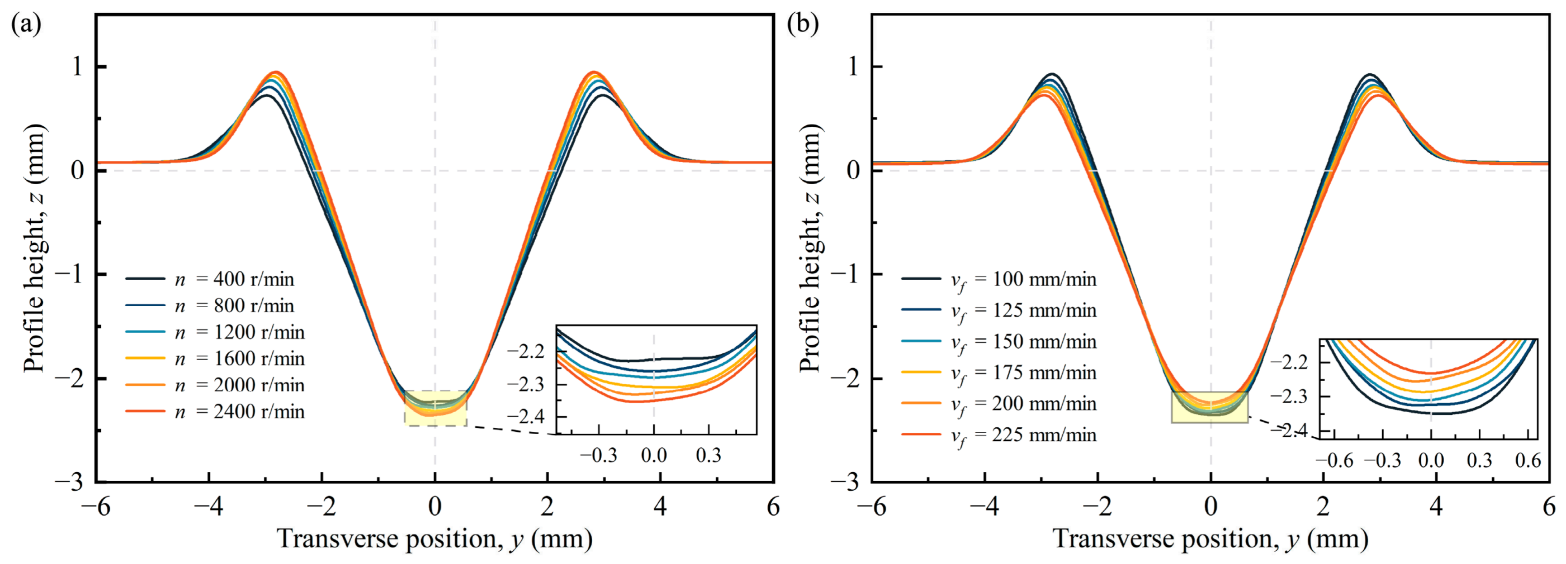
Effects of spindle speed and feed speed on the formed profiles predicted by the validated three-layer FE model: (**a**) spindle-speed effect at *v_f_* = 150 mm/min; (**b**) feed-speed effect at *n* = 1200 r/min.

The effects of spindle speed and feed speed on the final profile are further examined in Figure 24. Unlike the peak forming force, which reflects the local resistance during an individual engagement, the final profile is governed by the accumulation of plastic deformation over successive engagements. The nominal roll-beating depth is maintained at *d* = 2.37 mm for all cases. Figure 24a compares the predicted profiles at different spindle speeds with *v_f_* = 150 mm/min, while Figure 24b presents the corresponding results for different feed speeds at *n* = 1200 r/min.

As shown in Figure 24a, increasing the spindle speed from 400 to 2400 r/min produces a deeper tooth root and greater material pile-up on both sides of the groove. At a fixed feed speed, the spacing between successive engagements decreases as the spindle speed increases, which increases the overlap of adjacent deformation regions and promotes local plastic-deformation accumulation. The higher roller velocity also increases the local deformation rate and affects the material response during each engagement. The predicted profile variations are therefore associated with the combined influence of successive deformation accumulation and loading-rate-dependent material behavior.

Figure 24b shows that increasing *v_f_* from 100 to 225 mm/min produces a progressively shallower tooth root and less material pile-up on both sides of the groove. At a fixed spindle speed, the larger feed speed increases the spacing between successive engagements and reduces the overlap between adjacent deformation regions. Each material region therefore undergoes fewer repeated loading events and accumulates less plastic deformation. Although a larger engagement spacing increases the deformation required during an individual engagement, the reduced number of engagements per unit feed length results in lower overall deformation accumulation and a shallower final profile.

A higher spindle speed or lower feed speed promotes deformation accumulation, producing a deeper tooth root and greater material pile-up. This trend differs from that of the peak forming force discussed in Section 4.1. For example, increasing the feed speed raises the peak force while producing a shallower tooth root. The peak force therefore reflects the instantaneous deformation resistance during an individual engagement, whereas the final profile depends mainly on plastic deformation accumulated over successive engagements. The experimentally measured changes in tooth-root position exhibit trends consistent with the FE predictions under varying spindle speeds and feed speeds, as shown in Supplementary Figure S7.

### 4.3. Layerwise Deformation and Load Transfer Characteristics

**Figure 25:**
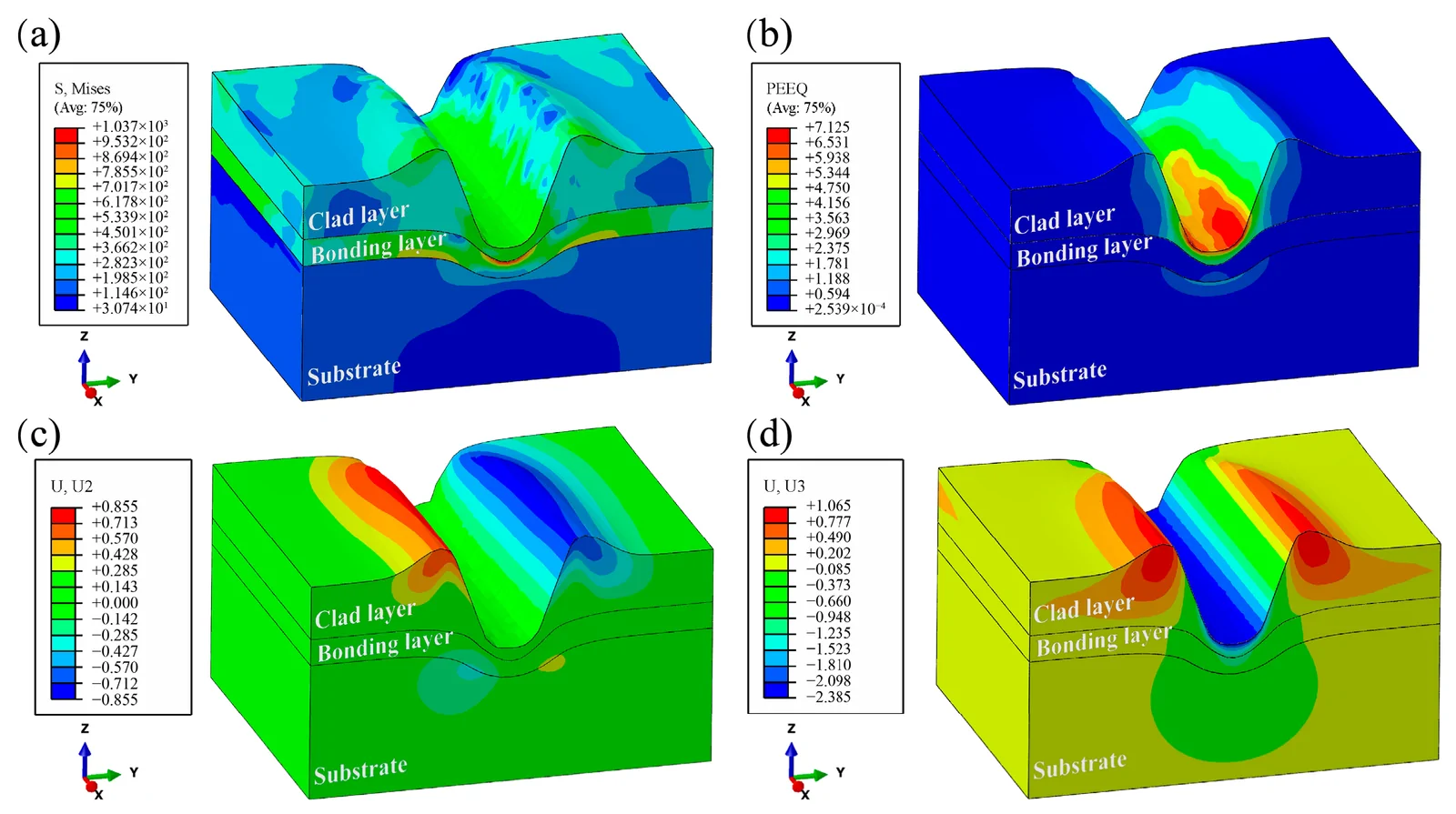
Stress, equivalent plastic strain, and displacement fields in the Fe/Al layered composite during cold roll-beating under a representative condition (*n* = 1200 r/min, *v_f_* = 150 mm/min, and *d* = 2.37 mm): (**a**) von Mises stress; (**b**) equivalent plastic strain (PEEQ); (**c**) transverse displacement U2; and (**d**) through-thickness displacement U3.

To examine the internal response of the Fe/Al layered composite during cold roll-beating, a representative condition of *n* = 1200 r/min, *v_f_* = 150 mm/min, and *d* = 2.37 mm is selected. Figure 25 presents the von Mises stress, equivalent plastic strain (PEEQ), transverse displacement U2, and through-thickness displacement U3 predicted by the three-layer FE model at the instant of maximum roller penetration during a representative engagement in the stable forming stage. These field variables are used to characterize load transfer through the layered structure and the associated development of local plastic flow. The model validation in Section 3.3 is based on experimental forming forces and final tooth profiles, while the local stress, plastic strain, and internal displacement fields have not been independently validated through direct measurements.

As shown in Figure 25a,b, the highest von Mises stresses are concentrated around the tooth root and the adjacent flank on the active contact side, extending from the steel clad through the equivalent bonding layer into the underlying aluminum substrate. Although the stress magnitude generally decreases with depth, the stress-affected region extends well beyond the zone of pronounced PEEQ. The stress field extends through a relatively large portion of the laminate, whereas pronounced plastic deformation remains concentrated near the region directly involved in profile formation. The PEEQ distribution shows that plastic strain is highest around the tooth root and adjacent flank and gradually decreases from the steel clad, through the equivalent bonding layer, into the aluminum substrate. Thus, the extent of load transmission is broader than that of the region undergoing significant plastic deformation.

The displacement fields in Figure 25c,d further clarify how localized plastic deformation develops into the characteristic features of the formed profile. During roller penetration, material beneath the tooth-root contact region is displaced predominantly in the negative *z*-direction. The resulting local compression drives material redistribution toward the surrounding regions, while the roller geometry and neighboring material constrain the available flow paths. A coupled displacement response therefore develops in the *y*-*z* plane: part of the material moves laterally along the *y*-direction toward both sides of the groove, while the adjacent free surface rises in the positive *z*-direction to form pile-up. This constrained material redistribution progressively generates the tooth root, flank transition regions, and material pile-up on both sides of the groove. The tooth root, flank transition geometry, and surface pile-up are therefore different geometric manifestations of the same localized material-redistribution process.

Comparison of the PEEQ and displacement fields shows that pronounced plastic strain remains highly localized around the tooth root and adjacent flank, whereas the U2 and U3 responses extend over a broader region that includes the equivalent bonding layer and the underlying aluminum substrate. This behavior indicates that localized plastic deformation in the roller-contact region is accompanied by a broader displacement response extending laterally and through the thickness under interlayer constraint. The final tooth geometry is formed by the combined action of local root penetration, lateral material flow, and progressive plastic-deformation accumulation over successive engagements.

## 5. Conclusions

This study addressed the challenge of representing the heterogeneous bonding region of explosively bonded Fe/Al layered composites in component-scale cold roll-beating simulations. An experimentally informed three-layer finite element framework was therefore developed by approximating the mechanically affected bonding region as a finite-thickness equivalent layer rather than an idealized zero-thickness interface.

The three-layer model gives better agreement with the experimental forming force and final profile than the simplified bilayer model. The mean absolute relative errors in forming force are 8.35% for the spindle-speed series and approximately 6.20% for the feed-speed series, while the mean profile NRMSE over six representative conditions is 3.27%. The additional material-property control simulations further indicate that the effective properties assigned to the bonding region influence both forming-force and final-profile predictions under the representative condition.

Spindle speed and feed speed affect the forming response through their influence on engagement spacing, repeated loading, and local deformation rate. Increasing spindle speed generally promotes plastic-deformation accumulation and produces a deeper tooth root and greater material pile-up, whereas increasing feed speed reduces deformation accumulation and results in a shallower profile. The peak forming force reflects the local deformation resistance during an individual engagement, while the final profile depends mainly on plastic deformation accumulated over successive engagements. The stress field extends beyond the region of pronounced plastic strain, and the displacement response spreads through a broader portion of the layered structure under interlayer constraint.

The present model treats the heterogeneous bonding region as a homogeneous equivalent layer and assumes perfect bonding between adjacent material domains. Interfacial damage, separation, and sliding are therefore not considered. Although the SEM and EDS observations reveal local microstructural and compositional heterogeneity, they do not establish the identities of specific Fe–Al reaction phases. Potential brittle fracture within the bonding region is also not explicitly represented in the model. Although the model has been validated against macroscopic forming forces and final profiles, the effects of local interfacial geometry and damage have not been independently quantified. In addition, although the equivalent bonding layer thickness was determined from the spatial envelope of multi-path microhardness measurements, its influence on the predicted forming force and final profile was not independently evaluated through a thickness sensitivity analysis. Future work may therefore consider variations in the equivalent layer thickness, explicitly wavy interfaces, cohesive-zone formulations, and graded descriptions of the bonding region.

## Figures and Tables

**Table 1:** EDS point-analysis results at locations A–D marked in Figure 5d,e (at.%).

Location	Fe	Al	C	Mn
Point A	48.12	50.03	1.26	0.18
Point B	44.37	53.62	1.70	0.29
Point C	34.79	63.47	1.49	0.23
Point D	50.21	46.93	1.52	0.55

Note: The listed values are semi-quantitative results obtained by EDS point analysis. Only the main detected elements are included.

**Table 2:** Johnson–Cook constitutive equations and material parameters for the steel clad, equivalent bonding layer, and aluminum substrate.

Material Region	Constitutive Equation	*E* (GPa)	*ν*	*ρ* (kg/m^3^)
Steel clad	$\sigma =(368.28+306.76\varepsilon^{0.31})[1+0.02\ln(\frac{{\dot{\varepsilon }}_{p}}{{\dot{\varepsilon }}_{0}})]$	210.00	0.28	7850
Equivalent bonding layer	$\sigma =(446.32+420.54\varepsilon^{0.28})[1+0.15\ln(\frac{{\dot{\varepsilon }}_{p}}{{\dot{\varepsilon }}_{0}})]$	185.76	0.25	5275
Al substrate	$\sigma =(87.28+40.13\varepsilon^{0.48})[1+0.19\ln(\frac{{\dot{\varepsilon }}_{p}}{{\dot{\varepsilon }}_{0}})]$	68.95	0.33	2700

Note: Thermal softening was neglected because the present model focuses on the mechanical and strain-rate-dependent responses under the investigated room-temperature forming conditions, for which thermal effects were assumed to be secondary [51]. The reference strain rate was defined as ${\dot{\varepsilon }}_{0}=10^{-3}s^{-1}$.

**Table 3:** Chemical compositions of the steel clad and aluminum substrate (wt.%).

Materials	C	Si	Mn	Ni	Fe	Cu	Zn	Cr	Al
Q235B steel	0.051	0.18	1.30	0.13	Bal.	0.12	—	0.03	—
1060Al	—	0.18	0.03	—	0.10	0.03	0.02	—	Bal.

**Table 4:** Process parameters used in the cold roll-beating experiments.

Varied Parameter	*n* (r/min)	*v_f_* (mm/min)	*d* (mm)
Spindle speed	400	150	2.37
	800	150	2.37
	1200	150	2.37
	1600	150	2.37
	2000	150	2.37
	2400	150	2.37
Feed speed	1200	100	2.37
	1200	125	2.37
	1200	150	2.37
	1200	175	2.37
	1200	200	2.37
	1200	225	2.37

**Table 5:** Quantitative comparison of experimental and FE-predicted forming forces under different processing conditions.

Variable	*n* (r/min)	*v_f_* (mm/min)	Experiment (Mean ± SD, kN)	Three-Layer FE (kN)	Error (%)	Bilayer FE (kN)	Error (%)
Spindle speed	400	150	12.58 ± 0.22	13.51	7.39	10.25	18.52
	800	150	12.89 ± 0.08	13.96	8.22	10.53	18.37
	1200	150	13.02 ± 0.15	14.00	7.54	10.80	17.05
	1600	150	12.56 ± 0.38	13.69	9.00	10.69	14.89
	2000	150	12.25 ± 0.17	13.22	7.92	10.39	15.18
	2400	150	11.84 ± 0.26	13.03	10.05	10.06	15.03
Mean error	—	—	—	—	8.35	—	16.51
Feed speed	1200	100	12.75 ± 0.23	13.62	6.82	10.74	15.76
	1200	125	12.86 ± 0.25	13.40	4.12	10.98	14.69
	1200	150	13.02± 0.15	14.00	7.54	10.80	17.05
	1200	175	13.30 ± 0.26	14.04	5.56	11.00	17.29
	1200	200	13.67 ± 0.31	14.68	7.39	11.67	14.63
	1200	225	14.16 ± 0.22	14.98	5.79	12.79	9.68
Mean error	—	—	—	—	6.20	—	14.85

**Table 6:** Comparison of profile-prediction errors between the three-layer and bilayer FE models.

Varied Parameter	Parameter Level	Three-Layer FE Model			Bilayer FE Model		
		Profile NRMSE (%)	Root-Depth Error (%)	Tooth-Crest Height Error (%)	Profile NRMSE (%)	Root-Depth Error (%)	Tooth-Crest Height Error (%)
Spindle speed	*n* = 400 r/min	3.30	4.40	3.38	5.34	12.94	10.03
	*n* = 1200 r/min	3.80	3.16	7.07	5.36	12.59	17.39
	*n* = 2400 r/min	3.65	2.42	6.85	5.33	16.56	12.12
Feed speed	*v_f_* = 100 mm/min	3.22	2.36	7.44	5.84	5.56	11.68
	*v_f_* = 175 mm/min	2.74	6.85	7.52	5.16	3.26	17.98
	*v_f_* = 225 mm/min	2.90	2.39	11.22	4.44	5.30	13.91
Mean		3.27	3.60	7.25	5.25	9.37	13.85

Note: For the spindle-speed cases, *v_f_* = 150 mm/min; for the feed-speed cases, *n* = 1200 r/min.

**Table 7:** Comparison of forming-force and final-profile predictions obtained from the original and material-property control models under the representative condition (*n* = 1200 r/min, *v_f_* = 150 mm/min, *d* = 2.37 mm).

Model	Material Properties of Bonding Region	Mean Peak Force (kN)	Force Error (%)	Profile NRMSE (%)
Experiment	—	13.02	—	—
Bilayer FE	No distinct bonding layer	10.80	−17.05	5.36
Three-layer FE	Identified equivalent properties	14.00	+7.54	3.80
Three-layer (steel)	Steel-clad properties	14.31	+9.91	4.20
Three-layer (Al)	Aluminum-substrate properties	10.36	−20.43	6.54

## Data Availability

The original contributions presented in this study are included in the article/Supplementary Materials. Further inquiries can be directed to the corresponding author.

## Supplementary Materials

The following supporting information can be downloaded at https://www.mdpi.com/article/10.3390/ma19214745/s1, Figure S1: Comparison of experimental measurements and inverse finite element predictions for nanoindentation: (a) indentation load–depth responses and (b) residual indentation surface profiles along the radial direction.; Figure S2: Representative comparison of experimental and FE-predicted true stress–true strain responses of the Fe/Al layered composite under SHPB loading at a strain rate of approximately 4.14 × 10^3^ s^−1^; Figure S3: Comparison of experimental and optimized FE load–displacement responses of the Fe/Al layered composite under quasi-static compression; Figure S4: Local sensitivity analysis of the equivalent bonding-layer strain-hardening parameters under quasi-static compression: effects of (a) A, (b) B, and (c) n on the predicted load–displacement responses. Each parameter was independently varied by ±10% and ±20% relative to its identified value, while the remaining parameters were held constant; Figure S5: Comparison of experimental and FE-predicted normal forming-force histories during a representative roller engagement for different friction coefficients (*μ* = 0.1, 0.2, and 0.3). Figure S6: Effect of the friction coefficient on final tooth-profile prediction: (a) comparison of experimental and FE-predicted tooth profiles and (b) variation in profile normalized root-mean-square error (NRMSE) with the friction coefficient. Figure S7: Comparison of experimentally measured and FE-predicted changes in tooth-root position under different (a) spindle-speed and (b) feed-speed conditions. The changes are calculated relative to the reference conditions of 400 r/min and 100 mm/min, respectively. Error bars indicate the spatial standard deviations obtained from five measurement positions within each specimen, without propagating the variability of the reference conditions. Table S1: Effect of the friction coefficient on the predicted mean peak forming force and final tooth-profile accuracy.
