# Supplementary material for: Parametric Finite Element Modeling of Cold Roll-Beating Forming of Explosively Bonded Fe/Al Layered Composites Incorporating an Equivalent Bonding Layer
Source: Materials (Basel). 2026 Nov 8;19(21):4745. doi: 10.3390/ma19214745 (PMC13765183; doi:10.3390/ma19214745)
Supplement: Supplementary file 1 [file materials-19-04745-s001.zip › materials-4633419-supplementary.pdf]

Supplementary Materials

S1. Supplementary Results for Constitutive Parameter Identification

Figures S1–S3 present comparisons between the experimental measurements and corresponding FE predictions used in the identification of the equivalent bonding-layer constitutive parameters. Figure S4 further illustrates the local sensitivity of the predicted quasi-static compression response to variations in the Johnson–Cook strain-hardening parameters *A*, *B*, and *n*.

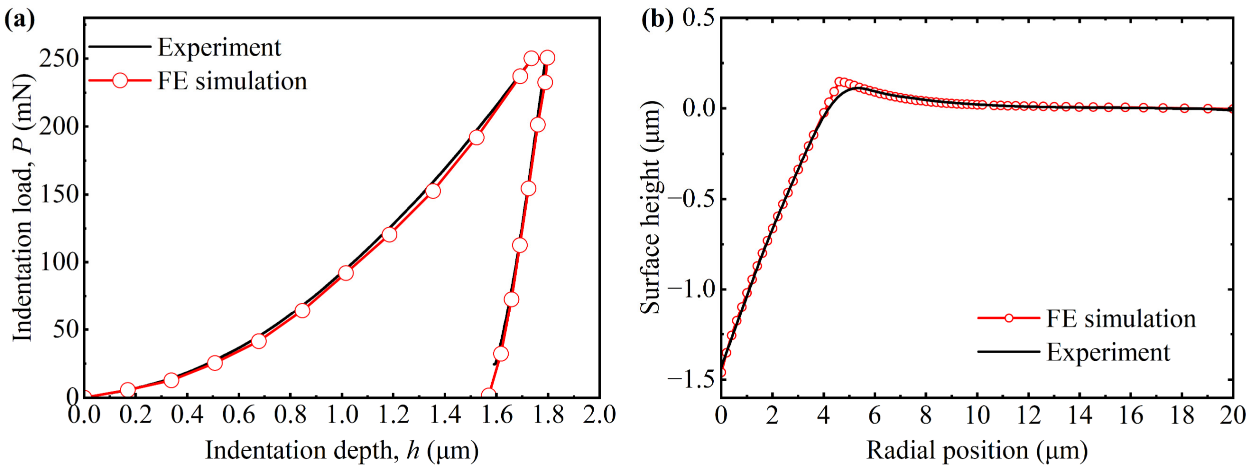

Figure S1. Comparison of experimental measurements and inverse finite element predictions for nanoindentation: (a) indentation load–depth responses and (b) residual indentation surface profiles along the radial direction.

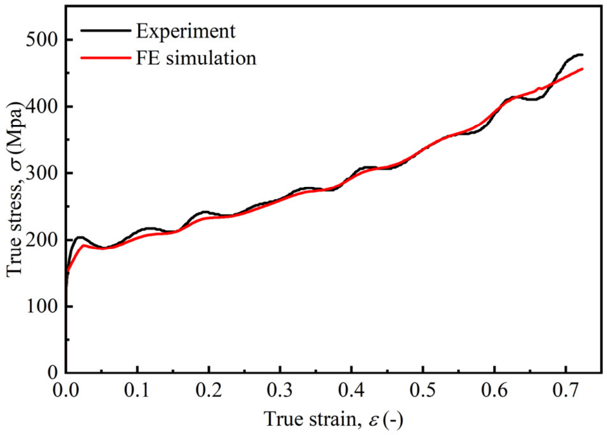

Figure S2. Representative comparison of experimental and FE-predicted true stress–true strain responses of the Fe/Al layered composite under SHPB loading at a strain rate of approximately 4.14×10^3 s^{-1}.

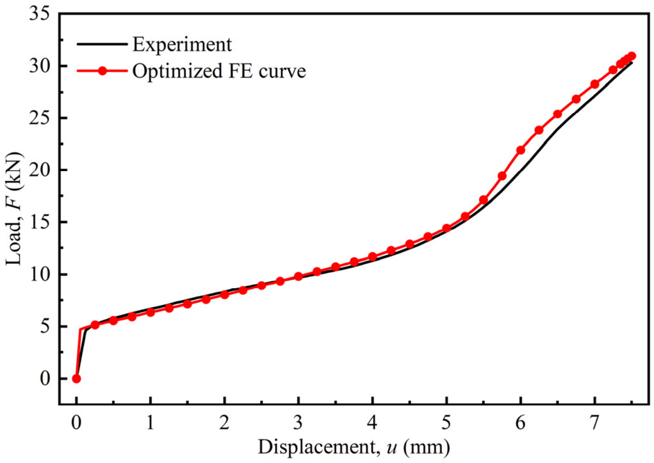

Figure S3. Comparison of experimental and optimized FE load–displacement responses of the Fe/Al layered composite under quasi-static compression.

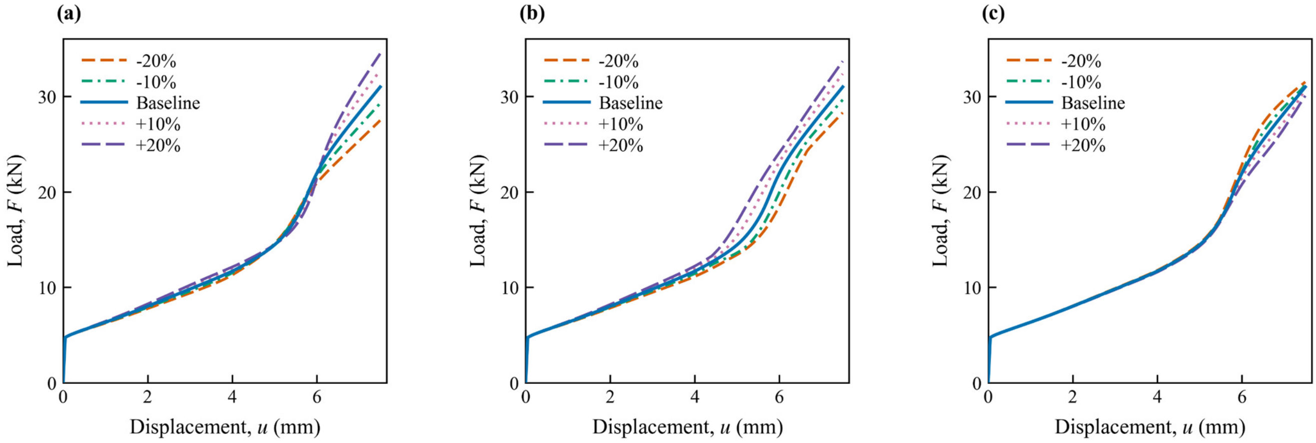

Figure S4. Local sensitivity analysis of the equivalent bonding-layer strain-hardening parameters under quasi-static compression: effects of (a) A, (b) B, and (c) n on the predicted load–displacement responses. Each parameter was independently varied by ±10% and ±20% relative to its identified value, while the remaining parameters were held constant.

S2. Sensitivity Analysis of the Friction Coefficient

To evaluate the influence of contact friction on the numerical predictions, additional simulations were performed with friction coefficients of 0.1, 0.2, and 0.3, while the remaining model parameters were kept unchanged. The predicted forming-force responses and final tooth profiles were compared with the experimental measurements under the representative forming condition of n = 1200 r/min, v_f = 150 mm/min, and d = 2.37 mm.

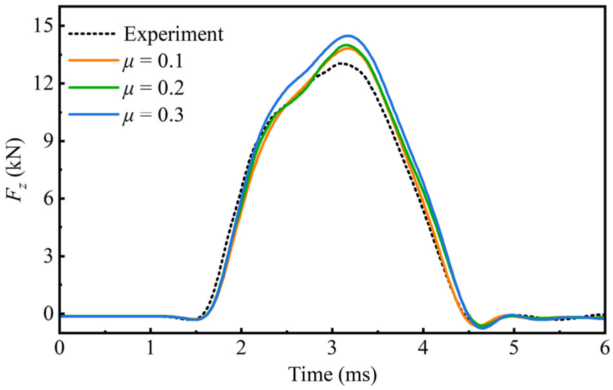

Figure S5. Comparison of experimental and FE-predicted normal forming-force histories during a representative roller engagement for different friction coefficients (μ = 0.1, 0.2, and 0.3).

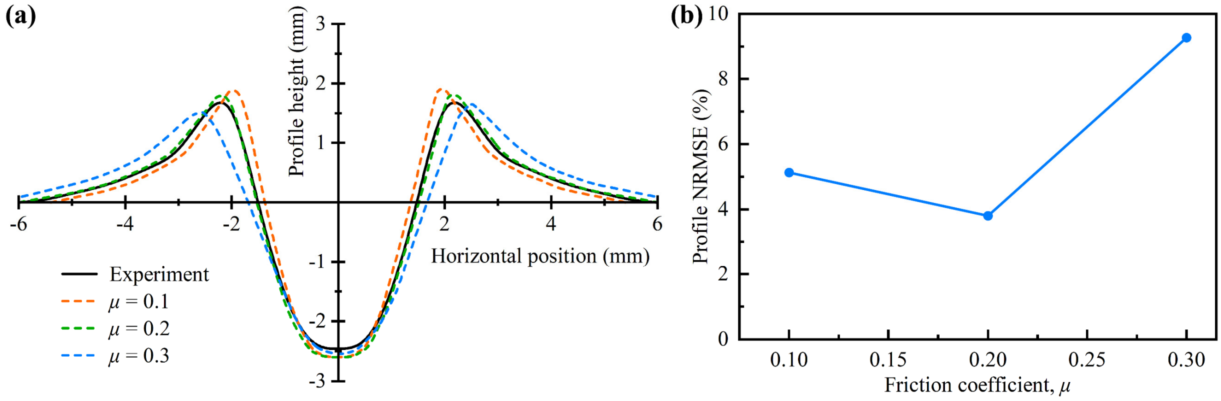

Figure S6. Effect of the friction coefficient on final tooth-profile prediction: (a) comparison of experimental and FE-predicted tooth profiles and (b) variation in profile normalized root-mean-square error (NRMSE) with the friction coefficient.

Table S1. Effect of the friction coefficient on the predicted mean peak forming force and final tooth-profile accuracy.

Friction coefficient, μ	FE peak force (kN)	Peak-force error (%)	Profile NRMSE (%)
0.1	13.97	+7.28	5.13
0.2	14.00	+7.54	3.80
0.3	14.15	+8.69	9.30

S3. Additional Comparison of Tooth-Root Position Changes

To supplement the final-profile validation presented in the main manuscript, experimentally measured changes in tooth-root position were compared with the corresponding FE predictions under different spindle-speed and feed-speed conditions. The comparisons are presented relative to the respective reference conditions in Figure S7.

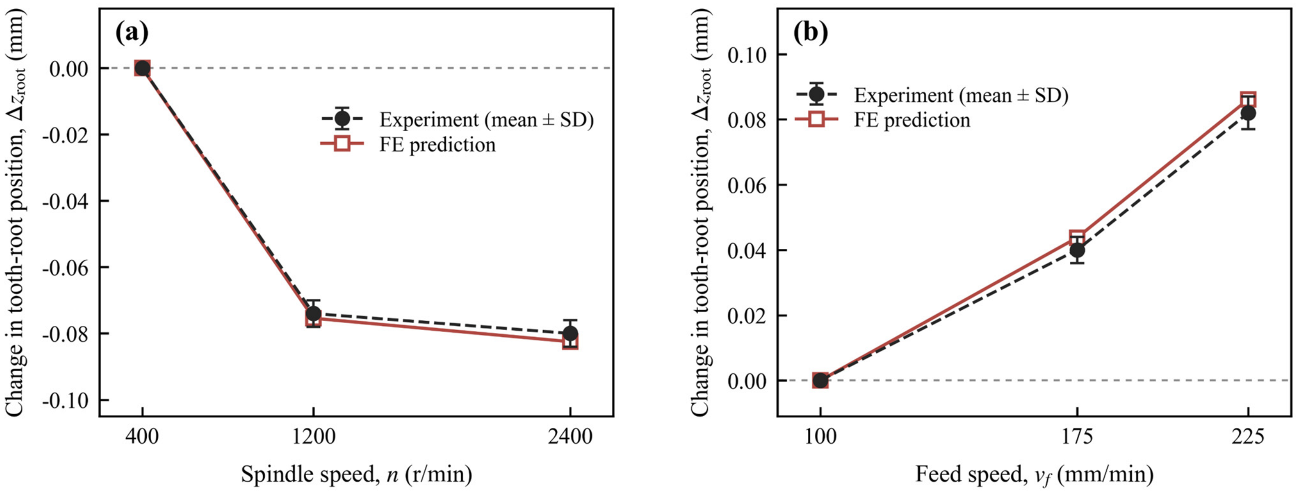

Figure S7. Comparison of experimentally measured and FE-predicted changes in tooth-root position under different (a) spindle-speed and (b) feed-speed conditions. The changes are calculated relative to the reference conditions of 400 r/min and 100 mm/min, respectively. Error bars indicate the spatial standard deviations obtained from five measurement positions within each specimen, without propagating the variability of the reference conditions.
